# Photobiomodulation at 830 nm with 5 J/cm^2^ does not promote PI3K/AKT/mTOR signalling pathway activation in hyperglycemic wounded cells

**DOI:** 10.1007/s10103-026-04859-8

**Published:** 2026-03-27

**Authors:** Patricia Kasowanjete, Sathish Sundar Dhilip Kumar, Nicolette Houreld

**Affiliations:** University of Johannesburg, Johannesburg, South Africa

**Keywords:** Diabetic foot ulcers, Diabetes mellitus, Wound healing, AKT, Chronic wounds, Photobiomodulation

## Abstract

The phosphatidylinositol 3-kinase (PI3K), protein kinase B (AKT), mammalian target of rapamycin (mTOR) (PI3K/AKT/mTOR) pathway regulates cell survival, proliferation, and the synthesis of protein. Photobiomodulation (PBM) stimulates biological processes by using low-powered light in the visible, red and near-infrared (NIR) spectrum. Despite its demonstrated efficacy, the cellular and molecular consequences, primarily on the PI3K/AKT/mTOR pathway, remain unclear. This study investigated PBMs impact on the PI3K/AKT/mTOR pathway in fibroblast (WS1) cells modelled as normal (N), wounded (W), hyperglycemic (D), hyperglycemic wounded (DW), and hyperglycemic hypoxic wounded (DHW). Non-irradiated cells served as controls. PBM was applied using an 830 nm diode at 5 J/cm^2^. Cellular changes at 24 and 48 h post-PBM were assessed via the Trypan blue exclusion assay (viability), light microscopy (morphology and migration), flow cytometry (proliferation and apoptosis), and the enzyme linked immunosorbent assay (ELISA) and western blot (VEGF, Bcl-2, PI3K, AKT, mTOR and GSK3β). PBM enhanced cellular migration rate, viability, and proliferation, without altering the PI3K/AKT/mTOR pathway. PBM at 830 nm with 5 J/cm^2^ promoted wound healing independently of the PI3K/AKT/mTOR pathway.

**Supplementary Information:**

The online version contains supplementary material available at 10.1007/s10103-026-04859-8.

## Introduction

Chronic wounds frequently develop in patients with diabetes mellitus (DM) due to impaired wound healing [1]. Anomalies related to abnormal fibroblast migration, proliferation, differentiation, and cell death have been linked to delayed wound healing. Studies also reveal that reduced cellular responses and hypoxia are important causes of delayed wound healing in person with diabetes [2]. Many biological processes essential to wound healing, including blood vessel formation, protein synthesis, and proliferation, depend on adequate oxygen supply [3]. Reversing the indisposition and incapacity of hyperglycemic wounds has been a slow process, with current available treatments, only 50% healing rate has been achieved and there is a 50% chance of wound recurrence [4]. Novel and effective treatment modalities for hyperglycemic wounds are necessary as the current therapies have a high relapse rate and their effects are transient [5].

Wound healing involves cellular and molecular processes to repair the damaged tissue. signalling pathways, growth factors and cytokines, regulate cell functions such as differentiation, proliferation, migration, and survival. These pathways interact at various levels, forming a unique network for specific growth factors, resulting in immediate or long-term biological effects [6, 7]. Wound healing is facilitated by different signalling pathways including the PI3K/AKT/mTOR pathway. PI3K/AKT/mTOR refers to an intracellular signalling pathway that controls cellular survival, metabolism, proliferation, growth, angiogenesis, cell cycle progression, apoptosis, and protein synthesis, and mediates the effects of vascular endothelial growth factor (VEGF). Activated AKT modulates downstream proteins such as mTORC1 and Glycogen Synthetase Kinase-3 beta (GSK3β), which has been linked to diseases like diabetes. An enzyme GSK3β is crucial for several physiological functions including, gene expression, glycogen metabolism and cellular signalling [8, 9].

Researchers are exploring novel strategies for chronic hyperglycemic wound healing, including dressings, tissue-engineered structures, pharmacological mediators, and non-invasive therapeutic techniques like photobiomodulation (PBM). Photobiomodulation therapy (PBMT) is a term that refers to a type of treatment based on PBM, and utilises photon energy at specific wavelengths and power densities [10]. PBM minimises pain, reduces inflammation and speedup wound healing [11, 12]. However, the specific underlying cellular and molecular mechanisms of action that creates the observed effects, remain incompletely understood. PBM parameters must be correctly selected to prevent the biphasic dosage response [13]. Abiphasic dose -response describes a pattern where a low dose have a minimal effect, a moderate dose promote efficacy, and high doses result to inhibition beyond a peak threshold [14]. PBMT is mostly performed using red light (620–700 nm) or near infrared (NIR) light (780–1270 nm), wavelengths known to interact with cellular systems through well characterised biological mechanisms. These wavelengths deliver energy at intensities that are non-damaging to tissue yet sufficient to stimulate key processes involved in repair and regeneration [14, 15]. The most well known way that PBM works, is based on the absorption of light by mitochondrial chromophores. The large number of mitochondria present in cells makes them respond favorably to absorbed light.

Numerous biological compounds, such as metalloproteins, oxyhaemoglobin, melanin, lipids and cytochrome c oxidase (COX) can interact with visible and NIR light [16]. The absorbed light increases the metabolic activity of the mitochondrial. Molecules inside the cell include adenosine triphosphate (ATP), which serves as a source of energy for cellular function; nitric oxide (NO), a vasodilator that promotes the activity of immune cells and oxygen transportation; calcium ions (Ca^2+^) and reactive oxygen species (ROS), which act as a secondary messengers and enhance transcription factors involved in wound repair and healing [17]. After the initial absorption of photon energy, cyclic adenosine monophosphate (cAMP), NO and Ca^2+^, ROS set off a number of signalling pathways that activate transcription factors. These transcription factors can increase the expression of genes related to protein synthesis, cellular migration and proliferation, anti-inflammatory signalling, anti-apoptotic proteins, and anti-oxidant enzymes [14]. The normalisation of the mitochondrial membrane potential in dysfunctional mitochondria lowers the formation of ROS and mitigates oxidative stress, whereas in normal cells, elevated mitochondrial membrane potential causes a transient increase in ROS [14]. PBM at 830 nm (NIR light) has been shown to increase the release of vascular endothelial growth factor (VEGF), activating the MAPK signalling pathway [18], facilitate epidermal growth factor (EGF) release and activation of the JAK/STAT signalling pathway [19], and significantly enhance fibroblast cell proliferation, viability, migration, and survival, through activation of the PI3K/AKT/mTOR signalling pathway [20]. Therefore this study was designed to see if 830 nm with 5 J/cm^2^ has the same effect on the PI3K/AKT/mTOR signalling as 660 nm with 5 J/cm^2^.

## Methodology

### Research design

This project was approved by the University of Johannesburg’s Faculty of Health Sciences Research Ethics Committee (clearance number, REC-1488-2022). This study made use of normal skin fibroblasts (WS1; procured from ATCC^®^, CRL-1502™, Manassas, VA, USA). Five cell models were employed: normal (N), wounded (W), hyperglycemic (D), hyperglycemic wounded (DW), and hyperglycemic hypoxic wounded (DHW).

A 10% supplement of fetal bovine serum (FBS; F9665 Sigma-Aldrich, Johannesburg, South Africa), was added to minimum essential media (MEM, M7278 Sigma-Aldrich, Johannesburg, South Africa). 1% penicillin (10,000 units)-streptomycin (10 mg/mL; P42942 Sigma-Aldrich, Johannesburg, South Africa), 1 mM sodium pyruvate (S8636 Sigma-Aldrich, Johannesburg, South Africa), 2 mM L-glutamine (D6429 Sigma-Aldrich, Johannesburg, South Africa), 0.1 mM non-essential amino acid (NEAA, 11140-035 Sigma-Aldrich, Johannesburg, South Africa), and 1% amphotericin B (250 µg/mL; A2942 Sigma-Aldrich, Johannesburg, South Africa) was used to grow the fibroblast cells. A hyperglycemic cell model was created by continuously culturing cells in MEM containing an extra 17 mM D-glucose. The basic media already contained 5.6 mM D-glucose, thus cells were cultured in a final glucose concentration of 22.6 mM [21–24]. A wounded cell model was achieved by performing a central scratch on a monolayer of WS1 cells (hyperglycemic or normal) seeded in 3.4 cm diameter tissue culture plates using a sterile 1 mL pipette. A hyperglycemic hypoxic wounded model was achieved by incubating hyperglycemic cells for 4 h under anaerobic conditions, and then wounded as described above [25]. Post-wounding, cells were incubated for 30 min before receiving PBM to give them time to adjust [19, 21, 22, 26].

### PBM experiments

**Fig. 1:**
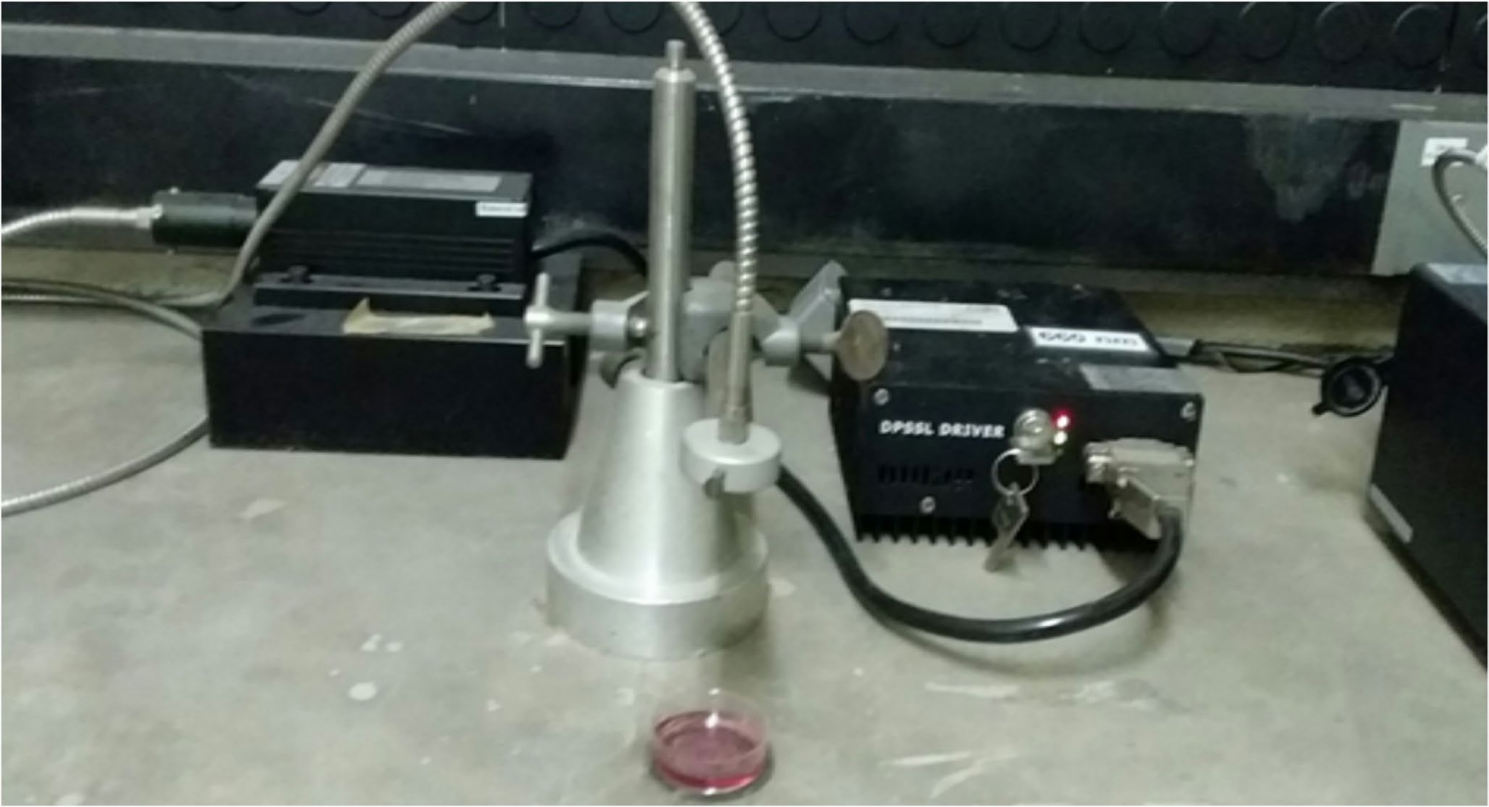
Experimental setup of a laser diode (PBM equipment) used for *in vitro* irradiation

For experiments, cells were seeded and exposed to PBM (in 3.4 cm diameter culture plates) at a density of 6 × 10^5^ or 1 × 10^6^ for flow cytometry. The experimental groups were exposed to a continuous wave near-infrared (NIR) diode laser with a wavelength of 830 nm and a power output of 100 mW (RGBlase, TE-CIRL-70G-830 SMA; Fremont, California). The Laser system was provided and installed by the Council for Scientific and Industrial Research (CSIR) – National Laser Centre (NLC) of South Africa. A fluence of 5 J/cm^2^ was used (spot size of 9.1 cm^2^; power density of 11 mW/cm^2^, and irradiation time of 7 min 34 s), which has been shown to induce favourable changes in WS1 cells in previous studies [19, 21, 23]. Control cells were sham irradiated (0 J/cm^2^). Normal (N) untreated cells (0 J/cm^2^) served as a baseline. PBM was administered to the cells in the dark to eliminate ambient light interference (Fig. 1). Thereafter, the cells were incubated at 37 °C in 5% CO_2_ for 24–48 h, after which the cellular responses were analysed.

### Cell morphology and migration rate

Cellular morphology and the rate of migration was assessed by inverted light microscopy (Olympus CKX41; Wirsam Scientific, Johannesburg, South Africa). The cellular migration rate towards the central scratch in wounded models was determined over a period of 48 h post-PBM and wound distances measured using the microscope imaging software (CellSens, version 2.3). Distance between wound margins was determined from three measurements (in the same plate, in three biological repeats) taken at different points along the ‘wound’ and at the identical location at 0, 24 and 48 h. The average distance (in µm) was used to calculate the migration rate in wounded, hyperglycemic wounded, and hyperglycemic hypoxic wounded cell models, and expressed in percentage (%) using the equation (At_0h_-At_time_)/At_0h_ x 100. In this equation At_0h_ is the average distance between wound margins at 0 h and At_time_ is the correspondent distance at various time points [27, 28].

### Proliferation, viability and apoptosis

#### Cellular proliferation

The BD Pharmingen™ BrdU FITC Flow Kit (559619/557891 BD Biosciences, the Scientific Group, Johannesburg, South Africa) was utilized to measure proliferation in all cell models. BrdU is an analogue of the DNA precursor thymidine that is integrated into the newly synthesized DNA as they enter and progress through the S phase of the cell cycle. Counting and classifying cells that are actively synthesizing DNA related to the cell cycle status (G0/G1, S, or G2/M phase) was made possible by the addition of 7-amino-actinomycin D (7-AAD), a DNA stain. This protocol was carried out in accordance with the manufacturer’s instructions. Cells (1 × 10^6^) were incubated for 24–48 h post-PBM. The culture medium was replaced with 1 mL of fresh complete media and incubated for an hour with 10 µM BrdU at 37 °C. Cells were detached with TrypLE™ Express (1 mL/25 cm^2^; 12563-029 Gibco^®^ Invitrogen™, ThermoFisher Scientific, Johannesburg, South Africa), thereafter the cells were washed twice with 1 mL of staining buffer and centrifuged at 300 x *g*. The pellet was placed on ice for 10 min after being re-suspended in BD Cytoperm™ Permeabilisation Buffer Plus. Cells were centrifuged and rinsed as previously described. Thereafter, the cells were re-suspended and incubated at 37 °C for 1 h in 300 µg/mL DNase in Dulbecco’s phosphate-buffered saline (DPBS). Cells were washed and re-suspended in 50 µL BD Perm/Wash™ Buffer containing fluorescent anti-BrdU (1:50 with 1X BD Perm/Wash Buffer) and left to stand at room temperature for 20 min. Thereafter, the Becton Dickinson (BD) Accuri C6 flow cytometer was used to perform cell analysis with a flow rate of 400 events per second, thereafter they were washed, centrifuged, and re-suspended in 7-ADD (20 µL in 1 mL staining buffer). A limit of 350 µL was set for each sample. Gating was performed based on the unstained control samples.

#### Viability

Cellular viability in all cell models was assessed using the Trypan blue exclusion assay following PBM. Equal amounts (10 µL) of 0.4% Trypan blue (T8154 Sigma-Aldrich, Johannesburg, South Africa) and cells were mixed and loaded into the Invitrogen Countess^®^ II FL automated bench top cell counter (ThermoFisher Scientific, Johannesburg, South Africa). The total number of unstained live cells (viable cells) and stained dead cells (non-viable cells) were counted and reported in percentage (%).

#### *Apoptosis (Annexin V/PI)*

In this study, the BD Pharmingen™ FITC Annexin V/PI Apoptosis detection kit II (556570 BD Biosciences, The Scientific Group, Johannesburg, South Africa) was used, following the manufacturer’s instructions, to determine the percentage of viable cells and those undergoing cell death (apoptosis and necrosis). In summary, post-PBM, detached cells were washed twice in cold phosphate-buffered saline (PBS), resuspended in binding buffer and incubated with 5 µL FITC Annexin-V and 5 µL propidium iodide for 15 min at room temperature in the dark. Cells were analysed on the BD Accuri C6 flow cytometer at a rate of 400 events per second with a limit set for each sample at 350 µL.

#### Human Bcl-2

Bcl-2 is an anti-apoptotic protein that prolongs cell life when it is expressed. The Bcl-2 In vitro-SimpleStep ELISA^®^ kit (ab202411, Abcam, Biocom Africa, Centurion, South Africa) was employed for the quantitative analysis of Bcl-2 in cells post-PBM. The assay was conducted following the manufacturer’s instructions. Cells were rinsed with PBS and dissolved in 250 µL 1× lysis buffer. After mixing, the cells were incubated at 4 °C with shaking for 30 min. The mixture was then centrifuged at 7571× *g* for 10 min at 4 °C and the supernatant was used for analysis. A 100 µL of standard/sample (cell lysate) was added to the pre-coated wells and incubated for 1 h. Cells were washed four times with wash buffer (Bio-Rad PW40 microplate washer). Thereafter, 100 µL of biotinylated anti-human Bcl-2 antibody was added and incubated for 1 h at room temperature. Cells were washed and incubated for 1 h in 100 µL HRP-conjugated streptavidin.

The cells were rinsed and incubated in the dark at room temperature with moderate shaking for 30 min in 100 µL of Tetramethylbenzidine (TMB) substrate. Color intensity was assessed using the VICTOR Nivo^®^ Multimode Plate Reader (Perkin-Elmer, Midrand, South Africa) at 450 nm after the reaction was stopped using 50 µL of stop solution. The average blank absorbance value was subtracted from the mean absorbance calculated for duplicate standards and samples. Sample concentration (pg/mL) was calculated from a standard curve using standards provided.

### PI3K/AKT/mTOR signalling proteins

#### *PI3K*,* p-AKT and p-mTOR*

In this study, the target signalling proteins (PI3K, p-AKT, and p-mTOR) were detected by western blot at 24 and 48 h post-PBM. Briefly, adherent cells were washed with ice-cold PBS, dislodged with a cell scraper, and subjected to centrifugation at 595 x g. for 5 min. The supernatant was removed and cells re-suspended in 200 µL ice-cold radioimmunoprecipitation assay (RIPA) buffer (9803, Cell signaling Technology^®^, Anatech Instruments (Pty) Ltd., Johannesburg, South Africa). Thereafter, 20 µL of 1 mM Phenylmethylsulphonyl (PMSF) protease (P-7626 Sigma-Aldrich, Johannesburg, South Africa) was added to 180 µL lysis buffer and incubated on ice for 30 min. Cells were then lysed through sonication, incubated at 4 °C for 10 min, and centrifuged (13,709 x g for 10 min). The supernatant (protein mix) was kept on ice and protein concentration quantified using the Pierce™ BCA protein assay kit (25225, Pierce, ThermoFisher Scientific, Randburg, South Africa). Laemmli buffer (2X; S3401, Sigma-Aldrich, Johannesburg, South Africa) was used as a diluent for the sample lysate to the loading protein concentration of 1 µg/µL and heated at 100^ο^C for 5 min. Protein samples (25 µL) was separated by sodium dodecyl sulfate polyacrylamide gel electrophoresis (SDS-PAGE) and transferred to an Immuno-Blot PVDF membrane (162 0177, Bio-Rad, Sandton, South Africa) for 3 h at 60 volts using a semi-dry blotter (Sigma-Aldrich, Johannesburg, South Africa, B2529). Blots were blocked for 10 min using bovine serum albumin (BSA- 5%) in Tris-buffered saline (TBS). After three cycles of washing, using Tween-20 (0.1%) in TBS, primary antibodies diluted 1:200 in BSA (SCBSC-8010, Santa Cruz Biotechnology, Anti-PI3-kinase p110 (D-4), mouse monoclonal antibodies, Anatech Instruments (Pty) Ltd., Johannesburg, South Africa; SCBSC-293133, anti-p-mTOR (59. Ser 2448); AB8932, anti-AKT1 (phospho S473), rabbit polyclonal, Biocom Africa (Pty) Ltd, Pretoria, South Africa; and sc-47724, GAPDH, Santa Cruz Biotechnology, Anatech Instruments (Pty) Ltd., Johannesburg, South Africa) was added and incubated overnight at 4 °C with gentle shaking. Thereafter, cells were washed three times (10 min each) in 0.1% Tween-20 in TBS. After washing, horseradish peroxidase secondary antibody (AP307P, Sigma-Aldrich, Johannesburg, South Africa, Goat anti-Rabbit IgG antibody, (H + L) HRP conjugate; or AP308P, Sigma-Aldrich, Johannesburg, South Africa, Goat Anti-Mouse IgG Antibody, (H + L) HRP conjugate) diluted 1:1,000 in PBS-T was added. This was followed by another wash in TBS. Thereafter, the membranes were incubated in 1% 3,3’-Diaminobenzidine (DAB) and 0.3% hydrogen peroxide in PBS for 5 min. The bands presence were scanned using a Bio-Rad ChemiDoc™ MP imaging system and Image lab 5.2.1. software. GAPDH served as a protein loading control.

#### VEGF

The Human VEGF ELISA Kit (Biocom Africa (Pty) Ltd, Pretoria, South Africa, Abcam ab100662) was used to quantitatively analyse the release of VEGF from cells into the culture media. The assay was conducted following the manufacturer’s instructions. Briefly, 100 µL of standards and samples (cell culture supernatant) were added to the pre-coated wells and incubated overnight at 4 °C with gentle shaking. Thereafter, the solution was discarded, and wells washed 4 times with 1x wash buffer (Bio-Rad PW40 microplate washer). Following this step, 100 µL of 1x biotinylated VEGF detection antibody was added and the mixture was incubated at room temperature for 1 h with gentle shaking. After washing, 100 µL of HRP-conjugated streptavidin secondary antibody was added and incubated for 45 min at room temperature with gentle shaking. After incubation, the solution was discarded, and plates washed as previously described. Thereafter, 100 µL of TMB substrate was added and incubated with moderate shaking for 30 min in the dark. Stop solution (50 µL) was then added. Absorbance measurements at 450 nm were performed using the VICTOR Nivo^®^ Multimode Plate Reader (Perkin-Elmer, Midrand, South Africa). The samples with unknown concentrations were determined in pg/mL from the standard curve after subtracting the background reading of all the unknown samples and standards.

#### GSK3β (Unphosphorylated and phosphorylated)

In this study, a solid phase sandwich ELISA kit (PathScan^®^ CST7265C, Total GSK-3 Beta Sandwich ELISA Kit; PathScan^®^ CST7311C, Phospho-GSK-3 Beta (Ser9) Sandwich ELISA Kit, Anatech Instruments (Pty) Ltd, Johannesburg, South Africa,) was used to analyse the endogenous concentrations of phosphorylated GSK3β (Ser9) and unphosphorylated GSK3β. Adherent cells were washed once with ice-cold PBS. Thereafter, 0.5 mL of 1X cell lysis buffer (ice-cold) (9803, Cell Signaling Technologies^®^, Anatech Instruments (Pty) Ltd, Johannesburg, South Africa), plus 1 mM PMSF was added and incubated for 5 min on ice. Adherent cells were scraped off using a cell scraper, after which the lysate was sonicated on ice and centrifuged for 10 min at 13,709 x g at 4 °C. After centrifuging, 100 µL cell supernatant (which is the lysate) was added to the microwells, and plates incubated for 2 h at 37 °C. Plates were washed four times with wash buffer (Bio-Rad, PW40 microplate washer), thereafter, 100 µL of reconstituted detection antibody was added and incubated for 1 h at 37 °C. Cells were washed again as previously described, followed by the addition of a 100 µL reconstituted secondary antibody (HRP-linked), and incubated at 37 °C for 30 min. Cells were washed as previously described, followed by the addition of 100 µL TMB substrate and incubated at 37 °C for 10 min. The reaction was then stopped by the addition of 100 µL stop solution and readings recorded at 450 nm using the VICTOR Nivo^®^ Multimode Plate Reader (Perkin-Elmer, Midrand, South Africa).

### Data analysis

SigmaPlot version 12.0 (Systat Software, Inc., California, USA) was used to analyse the results. PBM experiments were performed on three biological repeats (*n* = 3). The Student’s t test and Dunnett’s test were used to ascertain differences between groups. Differences were considered statistically significant when *P ≤ 0.05, **P ≤ 0.01, and ***P ≤ 0.001. The standard error of the mean (SEM) was used to express data. To determine the effect size for each independent sample, Cohen’s d (d Cohen) was used. Cohen’s d suggests that an effect size of less than 0.2 is insignificant, even if it is statistically significant; 0.5 is considered a medium effect size, while values larger than 0.8 are considered a large effect size.

## Results

### Cellular morphology and migration rate

**Fig. 2:**
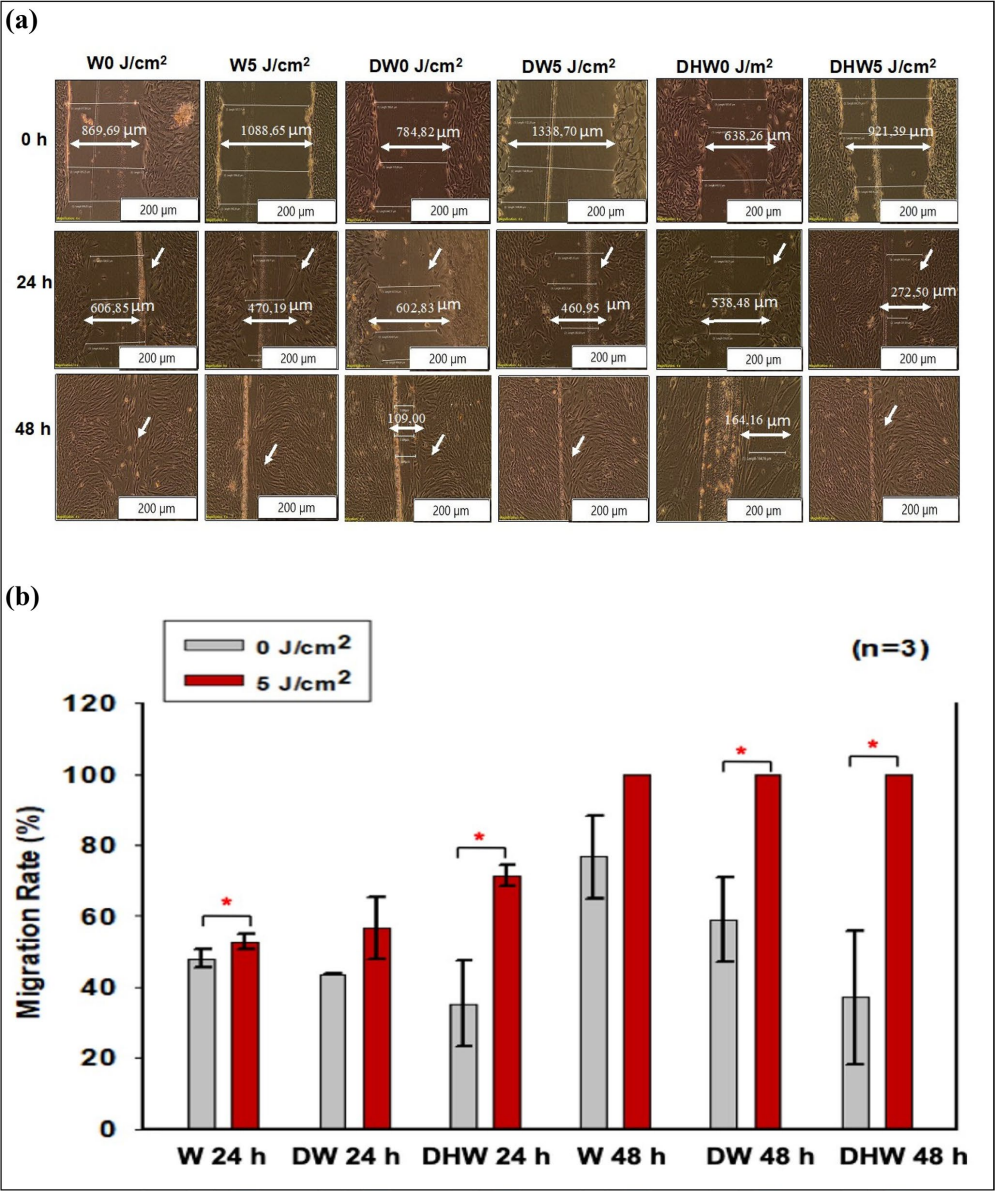
**a**) Micrography of cellular migration towards the central scratch observed at 0 h, 24 h and 48 h in both experimental (5 J/cm^2^) and control (0 J/cm^2^) cells in wounded (W), hyperglycemic wounded (DW) and hyperglycemic hypoxic wounded (DHW) models. The images show the central scratch on monolayer cells, mimicking a ‘wound’ as indicated by the arrows in the figures. There was increased cellular migration towards the central scratch in irradiated (5 J/cm^2^) W, DW and DHW cells. Magnification x200. **b**) Migration rate (%) in wounded (W), hyperglycemic wounded (DW), and hyperglycemic hypoxic wounded (DHW) cell models 24 and 48 h post-PBM at 830 nm. Significant probability compared to non-irradiated control cells is shown as * (*P < 0.05) (*SEM)

Post-PBM, changes in cellular morphology (in all models) were evaluated at 0, 24 and 48 h. Fibroblast cells appeared elongated, flat, slender, and spindle-shaped with projections protruding from the cell body (Fig. 2a). Experimental and control cell models exhibited no morphological changes at 0, 24 and 48 h post-PBM. The central scratch in wounded cell models produced a cell-free zone that resembled a “wound”, and the changes in morphology were defined by the directional motility of the cells, likely in response to chemotaxis and the surface compelled gradient of the extracellular matrix (ECM). An increase in cells along the wound edges at 24 and 48 h post-PBM (5 J/cm^2^) was seen. PBM’s effect on wounded cells was demonstrated by the fact that the central scratch was either completely or partially closed with new cells (Fig. 2a).

Migration rate in W, DW, and DHW cell models were assessed 24 and 48 h post-PBM. PBM significantly increased cellular migration rate (Fig. 2b). This was confirmed by complete wound closure (100% migration rate) at 48 h in irradiated cell models. There was an increase in migration rate at 24 h in irradiated W and DHW cell models *(P = 0.05)*, with a large effect size (d_Cohen_) of 1.5 and 3.5, respectively. At 48 h, a significant increase *(P = 0.05)* with large effect sizes (d_Cohen_) of 5.1 and 5.0, respectively, was observed in irradiated DW and DHW models.

There were no observable changes at 24 h when non-irradiated W cell models were compared to non-irradiated DW and DHW cell models, nor were any significant differences observed between non-irradiated DW and non-irradiated DHW cell models. The same comparisons were also made between irradiated W cells and irradiated DW and DHW cells. A significant increase was observed in irradiated DHW cells (*P = 0.007)*, while no significant change was observed in irradiated DW cells. When a comparison was made between non-irradiated W cell models and non-irradiated DW and DHW cell models at 48 h, a significant decrease in migration rate was observed in non-irradiated DW cells (*P = 0.005).* When irradiated W cells were compared to irradiated DW and DHW cells, no significant changes were observed.

### Cellular viability

**Fig. 3:**
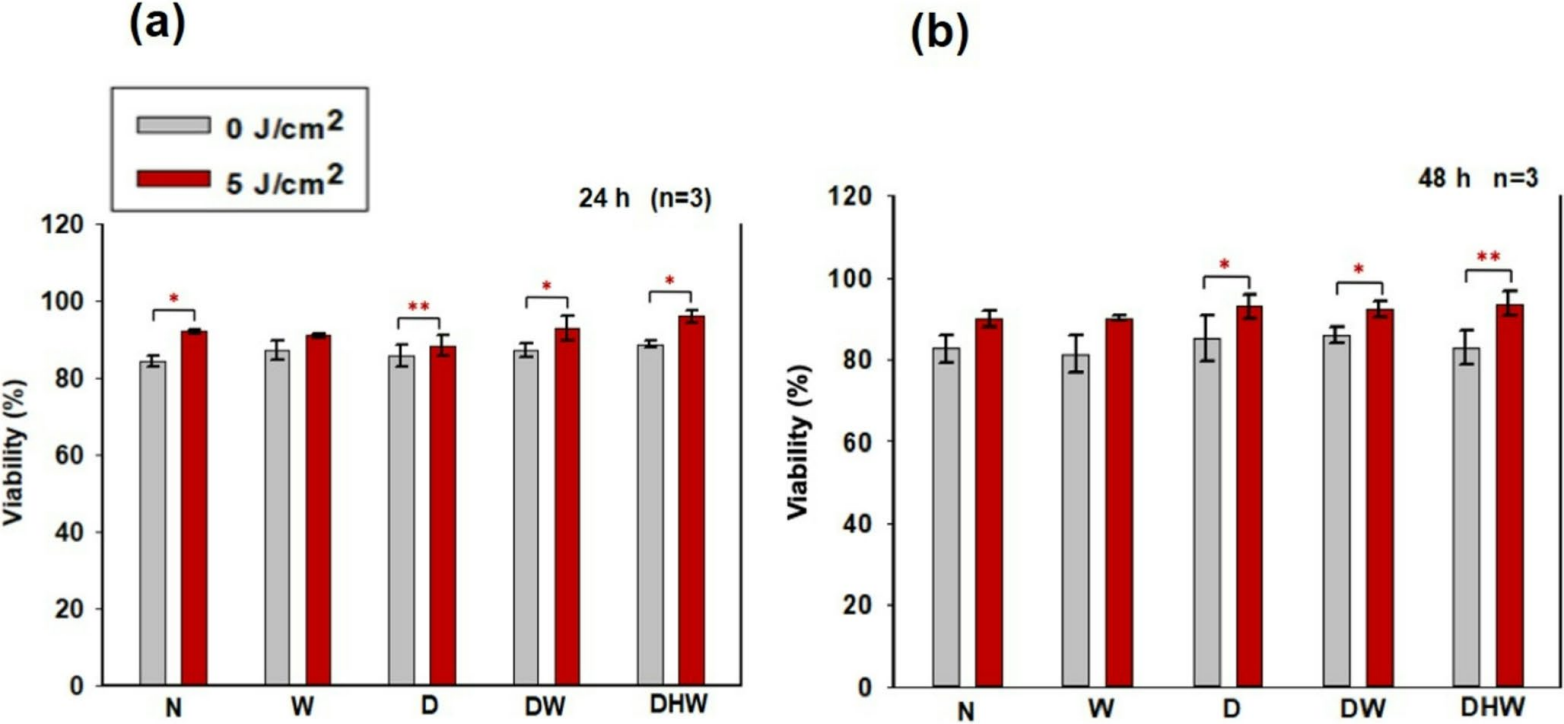
Cellular viability (%) in irradiated (5 J/cm^2^) and non-irradiated (0 J/cm^2^) normal (N), wounded (W), hyperglycemic (D), hyperglycemic wounded (DW), and hyperglycemic hypoxic wounded (DHW) cell models 24 h (**a**) and 48 h (**b**) post-PBM as determined by the Trypan blue exclusion assay. Significant probability is indicated as *P < 0.05 and **P < 0.01 (SEM)

Cellular viability was assessed using the Trypan blue exclusion assay 24 and 48 h post-PBM (Fig. 3). A significant increase in viability at 24 h was observed in N, D, DW, and DHW cell models (*P = 0.02*,*P = 0.01, P = 0.03*, and *P* = 0.03, respectively), with medium to large effect sizes (d_Cohen_ of 5.3, 0.5, 1.7, and 4.7 respectively) (Fig. 3a). While at 48 h (Fig. 3b), a significant increase was seen in D, DW, and DHW cell models *(P = 0.05, P = 0.02*, and *P* = 0.01, respectively), all with large effect sizes (d_Cohen_ of 1.3, 2.0, and 2.2, respectively). Further comparisons were made between non-irradiated N cell models and non-irradiated and irradiated W, D, DW and DHW cell models. At 24 h, a significant increase in viability was observed in non-irradiated W cell models (*P = 0.010)* and irradiated DHW cell models *(P = 0.038).* No observable changes were seen at 48 h.

### Proliferation

Cell proliferation was assessed using BrdU incorporation during the S phase of the cell cycle and analysed by flow cytometry (Table 1). At 24 h, there was a decrease in cells going through the synthesis phase (S phase) in irradiated W cell models *(P = 0.03)*, with a small effect size (d_Cohen_ of 0.4). Irradiated N cell models showed a significant decrease in the percentage of cells in G_0_/G_1_ *(P = 0.04*, with a large effect size (d_Cohen_) of 3.4), and an increase in cells in G_2_/M (*P = 0.008*, with an effect size (d_Cohen_) of 4.9). At 48 h post-PBM, a decrease in cells progressing through the S phase was observed in W cells (*P = 0.02)* with a large effect size (d_Cohen_) of 2.2, while an increase was seen in D (*P = 0.05*, with an effect size (d_Cohen_) of 0.2), DW *(P = 0.01*, with an effect size (d_Cohen_) of 0.4), and DHW *(P = 0.03*, with an effect size (d_Cohen_) of 2.1) cell models (Table 1). An increase in cells in the resting phase (G_0_/G_1_) was observed in W cell models (*P = 0.03)*, with a large effect size (d_Cohen_) of 1.3). A significant decrease was observed in D *(P = 0.03*, with a large effect size (d_Cohen_) of 1.5), DW *(P = 0.03*, with a large effect size (d_Cohen_) of 0.9), and DHW (*P = 0.04*, with an effect size (d_Cohen_) of 1.4) cell models. There was also an increase in cells in the mitotic phase (G_2_/M) in N (*P = 0.05*, with a medium effect size (d_Cohen_) of 0.5) and W (*P = 0.01*, with a large effect size (d_Cohen_) of 1.7) cell models.

**Table 1:** Cellular proliferation in non-irradiated (0 J/cm^2^) and irradiated (5 J/cm^2^) normal (N), wounded (W), hyperglycemic (D), hyperglycemic wounded (DW), and hyperglycemic hypoxic wounded (DHW) cell models as analysed by flow cytometry (5-bromo-2′-deoxyuridine (BrdU) incorporation and 7-aminoactinomycin D (7-AAD) at 24 and 48 h post-PBM at 830 nm. Significant probability compared to untreated controls is indicated as *P ≤ 0.05 and **P ≤ 0.01 (SEM)

		G0/G1 Phase (%)			S Phase (%)			G2/M Phase (%)	
		0 J/cm^2^	5 J/cm^2^		0 J/cm^2^	5 J/cm^2^		0 J/cm^2^	5 J/cm^2^
**24 h**	**N**	32 ± 3	17 ± 4*		9 ± 3	3 ± 2		41 ± 5	67 ± 6*
	**W**	42 ± 4	45 ± 4		5 ± 2	4 ± 1*		40 ± 9	34 ± 8
	**D**	28 ± 5	21 ± 5		7 ± 1	16 ± 6		50 ± 3	44 ± 2
	**DW**	29 ± 3	30 ± 3		2 ± 1	4 ± 2		48 ± 9	54 ± 7
	**DHW**	27 ± 2	35 ± 5		7 ± 2	4 ± 2		54 ± 14	45 ± 2
**48 h**	**N**	33 ± 4	33 ± 7		24 ± 9	17 ± 8		7 ± 8	12 ± 11
	**W**	35 ± 10	45 ± 5*		17 ± 6	7 ± 3*		13 ± 5	24 ± 8
	**D**	47 ± 7	27 ± 20*		8 ± 4	13 ± 7*		14 ± 8	10 ± 2
	**DW**	46 ± 10	38 ± 7*		12 ± 5	14 ± 5**		6 ± 3	10 ± 8
	**DHW**	47 ± 6	33 ± 13*		9 ± 4	17 ± 8*		12 ± 8	3 ± 2

Further comparisons were made between non-irradiated N cell models and non-irradiated and irradiated W, D, DW, and DHW cell models. At 24 h, a significant increase in cells in the resting phase (G_0_/G_1_) was observed in non-irradiated W (*P = 0.001*), D (*P = 0.010*), DW (*P = 0.002*), and DHW (*P ≤ 0.001*) cell models, as well as in irradiated W (*P = 0.001*), DW (*P = 0.011*), and DHW (*P = 0.002*) cell models. No significant changes in cells progressing through the synthesis phase (S phase) were observed between any of the models. There was a significant increase in cells in the G_2_/M phase in non-irradiated D cell models (*P = 0.044*).

At 48 h, when comparing non-irradiated N cell models to non-irradiated and irradiated W, D, DW, and DHW cell models, a significant increase in cells in the G_0_/G_1_ phase was observed in non-irradiated D (*P = 0.038*) and DHW (*P = 0.023*) cell models, and irradiated W cell models (*P = 0.027*). A significant decrease in cells progressing through the S phase was observed in non-irradiated W *(P = 0.014)*, D *(P = 0.002*), DW *(P = 0.007)*, and DHW (*P = 0.002)* cell models, as well as in irradiated D (*P = 0.019*) and DW (*P = 0.014)* cell models. No significant differences in the population of cells in the G_2_/M phase between models was observed.

### Apoptosis

#### Annexin V/PI

Cellular survival in all cell models (N, W, DW, and DHW cells) was analysed using the Annexin V/PI flow cytometry kit at both 24 and 48 h post-PBM. No significant differences in the percentage of viable cells, early apoptosis, and late apoptosis were observed at 24 h (Table 2). A notable increase in necrotic cells was observed in irradiated DHW cell models *(P = 0.04)* with a large effect size (d_Cohen_ of 3.0) at 24 h. At 48 h post-PBM, a significant increase in viable cells were observed in DHW cell models *(P = 0.04)* with a large effect size (d_Cohen_ of 12.2) (Table 2).

**Table 2:** Cellular changes observed in viable cells, early and late apoptosis, and dead cells (necrotic cells) 24 and 48 h post-PBM at 830 nm with 5 J/cm^2^ using Annexin V/PI in normal (N), wounded (W), hyperglycemic (D), hyperglycemic wounded (DW), and hyperglycemic hypoxic wounded (DHW) cells (*n* = 3). Significant probability as compared to untreated control cells is shown as * *P* ≤ 0.05 (± SEM)

			Viability (%)		Early Apoptosis (%)			Late apoptosis (%)		Necrosis (%(%)	
		0 J/cm^2^		5 J/cm^2^		0 J/cm^2^	5 J/cm^2^	J/cm^2^	5 J/cm^2^	0 J/cm^2^	5 J/cm^2^
**24 h**	**N**	67 ± 13		67 ± 10		26 ± 9	24 ± 7	10 ± 1	10 ± 3	0.2 ± 0.2	0.1 ± 0.1
	**W**	67 ± 15		68 ± 12		22 ± 9	17 ± 10	13 ± 6	12 ± 3	0.2 ± 0.3	0.2 ± 0.3
	**D**	74 ± 10		68 ± 10		21 ± 9	16 ± 14	10 ± 3	12 ± 6	0.1 ± 0.1	0.1 ± 0.0
	**DW**	69 ± 15		71 ± 13		17 ± 10	21 ± 9	13 ± 7	11 ± 2	0.3 ± 0.1	0.2 ± 0.1
	**DHW**	70 ± 18		80 ± 12		18 ± 14	16 ± 14	16 ± 11	12 ± 4	0.2 ± 0.1	1.4 ± 0.7*
**48 h**	**N**	84 ± 1		79 ± 4		17 ± 6	21 ± 5	1.7 ± 2	1.5 ± 1	0.2 ± 0.1	0.2 ± 0.1
	**W**	84 ± 3		84 ± 0		14 ± 3	17 ± 7	1.7 ± 2	1.9 ± 2	0.2 ± 0.1	0.2 ± 0.0
	**D**	80 ± 4		81 ± 3		18 ± 1	20 ± 6	1.7 ± 1	1.7 ± 1	0.1 ± 0.0	0.1 ± 0.1
	**DW**	79 ± 2		84 ± 1		20 ± 7	17 ± 7	3.4 ± 2	4.9 ± 6	0.3 ± 0.1	0.3 ± 0.1
	**DHW**	78 ± 2		97 ± 1*		17 ± 1	9 ± 10	3.6 ± 2	1.7 ± 2	0.2 ± 0.0	0.8 ± 0.6

Further comparisons were made between non-irradiated N cell models and non-irradiated and irradiated W, D, DW, and DHW cell models. At 24 h post-PBM, no significant change was observed in viable cells in the non-irradiated models, while a significant decrease was observed in irradiated DHW cells *(P = 0.021).* No significant differences were observed in early and late apoptosis, as well as in necrotic cells. Similarly, comparisons were made at 48 h between non-irradiated N cell models and non-irradiated and irradiated W, D, DW, and DHW cell models. A significant decrease in viable cells was observed in non-irradiated DHW cell models (*P = 0.042*), while an increase was observed in irradiated DHW cell models (*P = 0.001*). No significant changes were observed in early and late apoptosis, and necrosis.

#### Human Bcl-2

**Fig. 4:**
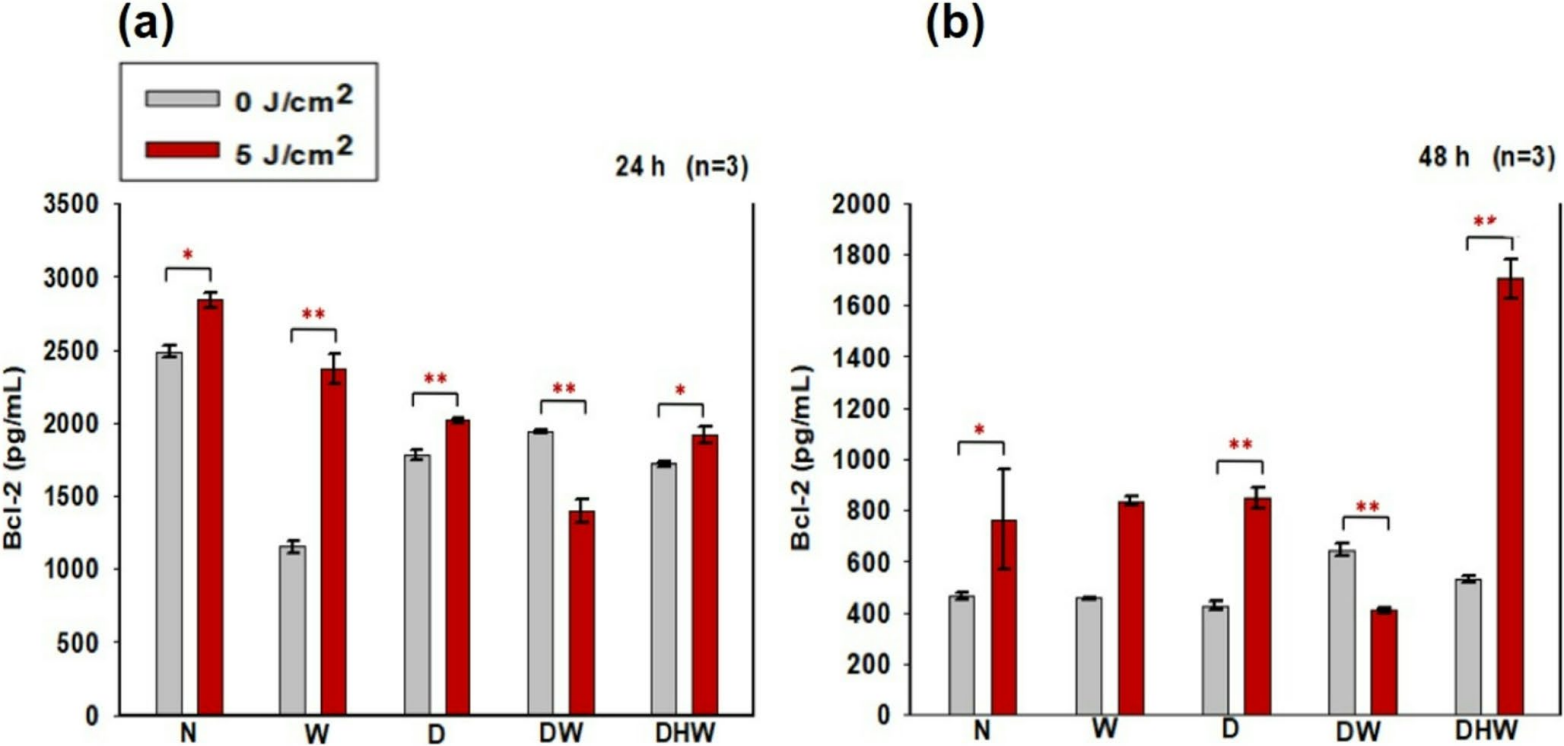
Bcl-2 was determined in non-irradiated (0 J/cm^2^) and irradiated (5 J/cm^2^) Normal (N), wounded (W) hyperglycemic (D), hyperglycemic wounded (DW), and hyperglycemic hypoxic wounded (DHW) cells at 24 h (**a**) and 48 h (**b**) post-PBM at a wavelength of 830 nm (*n* = 3). Significant probability is shown as (P < 0.05*) and P < 0.01** (SEM)

The effects of PBM at 830 nm and a fluence of 5 J/cm^2^ on apoptosis in hyperglycemic wound healing were also assessed via intracellular Bcl-2 using ELISA (Fig. 4). There was a notable increase in Bcl-2 at 24 h in irradiated N (*P = 0.022* with a large effect size (d_Cohen_) of 2.0), W (*P = 0.006* with a large effect size (d_Cohen_) of 70.0), D *(P = 0.005* with a large effect size (d_Cohen_) of 24.0), and DHW *(P = 0.035)* with a large effect size (d_Cohen_) of 2.7) cell models, while DW models showed a significant decrease *(P = 0.007* large effect size (d_Cohen_) of 18.7) (Fig. 4a). At 48 h, a significant increase in Bcl-2 was observed in N *(P* = 0.048), D *(P = 0.002)*, and DHW *(P = 0.002*) cell models with large effect sizes (d_Cohen_ of 22.0, 8.4, and 8.1, respectively), while a significant decrease was noted in DW cell models *(P = 0.003)* with a large effect size (d_Cohen_) of 15.6) (Fig. 4b).

Further comparisons were made between non-irradiated N cell models and non-irradiated and irradiated W, D, DW, and DHW cell models. At 24 h post-PBM a significant decrease in the secretion of Bcl-2 was noted in non-irradiated W, D, DW, and DHW models *(P = 0.001)*. At 48 h, a significant increase was observed in non-irradiated DW cell models (*P = 0.033)*, with in significant difference observed in the other cell models.

### VEGF

**Fig. 5:**
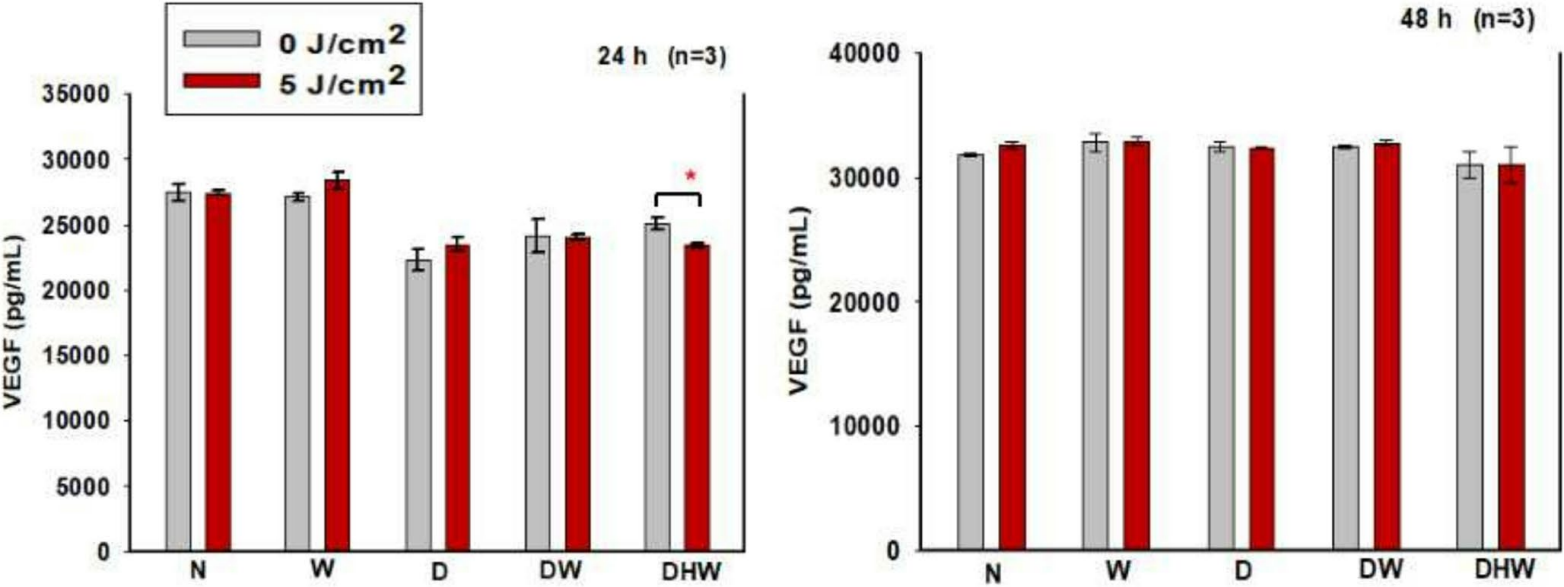
Vascular endothelial growth factor (VEGF) in normal (N), wounded (W), hyperglycemic (D), hyperglycemic wounded (DW), and hypoxic hyperglycemic wounded (DHW) cell models at 24 h (**a**) and 48 h (**b**) post-PBM at 830 nm with 5 J/cm^2^ (*n* = 3). Significant probability is shown as *P ≤ 0.05 (SEM)

Fibroblasts are among the various cells that take part in the wound healing process. Growth factors such as VEGF, which are produced by fibroblasts, are important for angiogenesis, collagen deposition, and epithelisation, all of which are aspects of wound healing. Additionally, it promotes vascular porousness, serves as a chemotactic agent, and is a mitogen for endothelial cell [29]. This study measured VEGF 24 h and 48 h post-PBM using ELISA (Fig. 5). The only significant change observed was at 24 h in DHW cells (*P < 0.05)* with a large effect size (d_Cohen_ of 3.0) (Fig. 5a). No significant change was seen 48 h post - PBM (Fig. 5b). This study compared non-irradiated N cells to non-irradiated and irradiated W, D, DW, and DHW cells using one-way ANOVA 24 h post-PBM. A significant decrease in VEGF was seen in DHW cells *(P = 0.053)*, and no observable change was seen in W, D, and DW cells. A significant decrease was seen in irradiated D *(P = 0.002)*, DW (*P = 0.050*), and DHW cells *(P = 0.041*) while at 48 h post - PBM, when the same models were compared (non-irradiated N cells were compared to non-irradiated W, D, DW and DHW cells), there was no significant changes observed, while a significant increase in VEGF was observed in irradiated D cells *(P = 0.002*) compared to irradiated N cells.

### PI3K, p-AKT, p-mTOR and GSK3β

This study evaluated PI3K, activated (p-) AKT1, p-mTOR, GSK3β, and p-GSK3β, using western blot and ELISA to determine the activation of the PI3K/AKT/mTOR pathway and downstream GSK3β post-PBM.

#### Western blot (PI3K, p-AKT1 and p-mTOR)

**Fig. 6:**
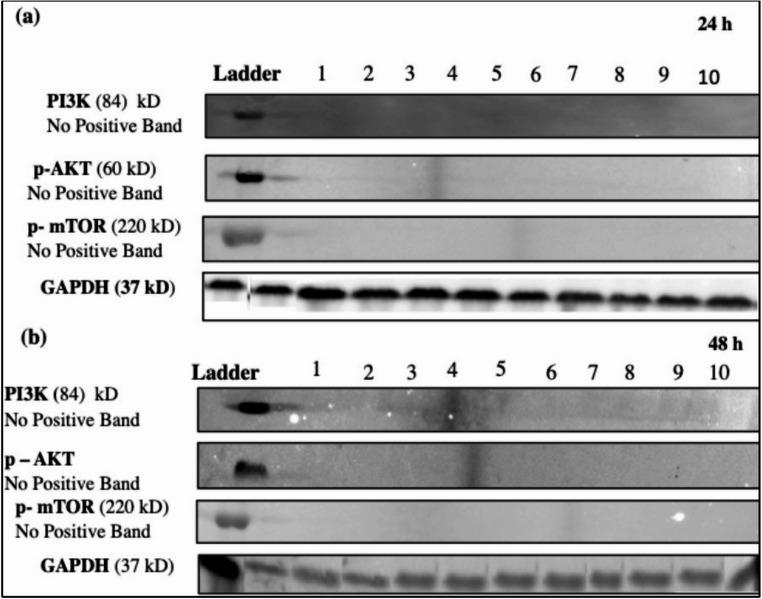
Analysed western blot for PI3K, p-AKT, and p-mTOR in non-irradiated (0 J/cm^2^) and irradiated (5 J/cm^2^) normal (N), wounded (W), hyperglycemic (D), hyperglycemic wounded (DW), and hyperglycemic hypoxic wounded (DHW) cells at 24 h (**a**) and 48 h (**b**) following irradiation at 830 nm. GAPDH served as a loading control. Lane 1 - N 0 J/cm^2^; Lane 2 - N 5 J/cm^2^; Lane 3 - W 0 J/cm^2^; Lane 4 - W 5 J/cm^2^; Lane 5 - D 0 J/cm^2^; Lane 6 - D 5 J/cm^2^; Lane 7 - DW 0 J/cm^2^; Lane 8 - DW 5 J/cm^2^; Lane 9 -DHW 0 J/cm^2^; and Lane 10 - DHW 5 J/cm^2^

Western blotting was used to detect PI3K, p-AKT1 and p-mTOR at 830 nm in both non-irradiated and irradiated N, W, D, DW, and DHW cell models at both 24 h and 48 h following PBM (Fig. 6). GAPDH was used as a positive loading control. PI3K, p-AKT1, and p-mTOR did not show any positive bands (protein presence) 24 h (Fig. 6a) or 48 h post-PBM (Fig. 6b).

#### ELISA (Total GSK3β and phosphorylated (p-)GSK3β)

**Fig. 7:**
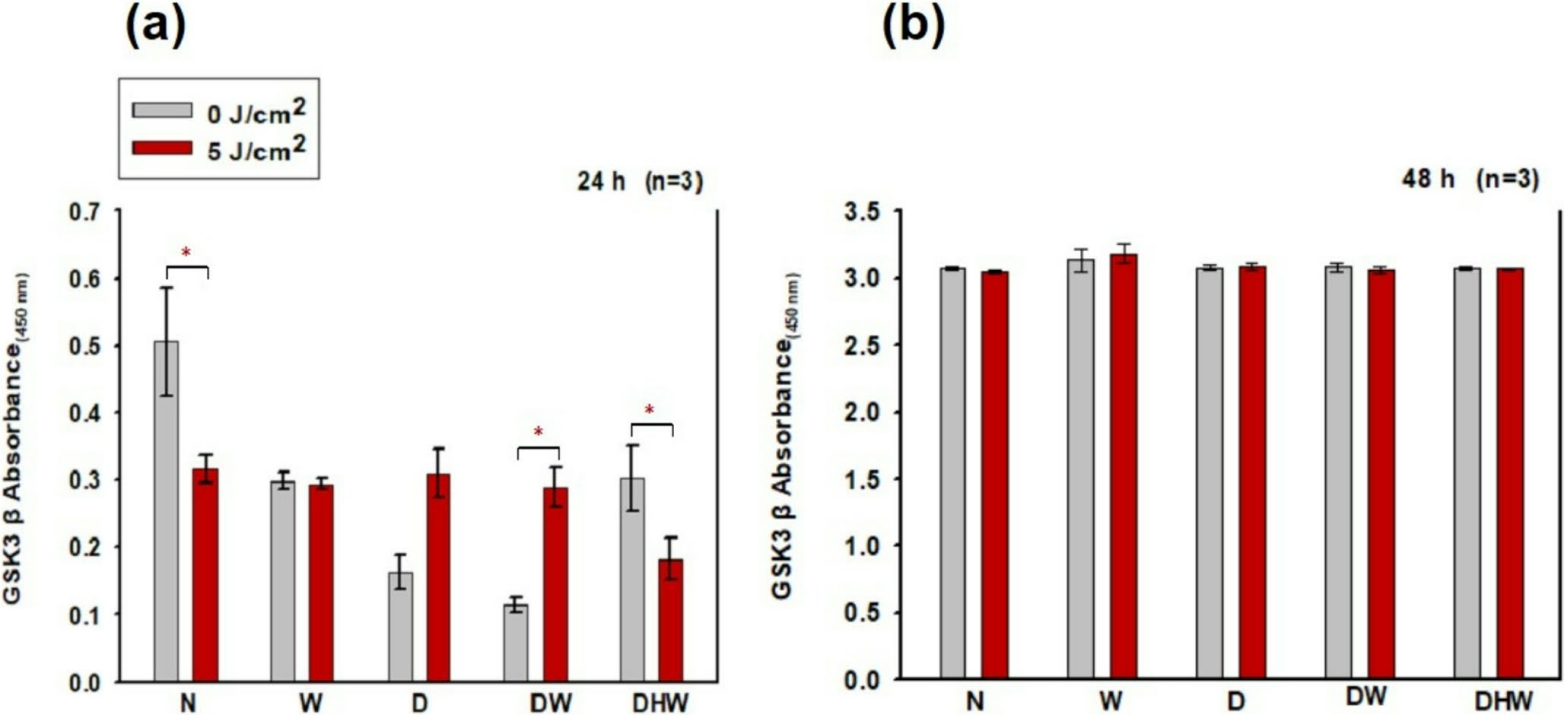
Total GSK3β in normal (N), wounded (W), hyperglycemic (D), hyperglycemic wounded (DW), and hyperglycemic hypoxic wounded (DHW) cell models at 24 h (**a**) and 48 h (**b**) post-PBM at 830 nm with 5 J/cm^2^ (*n* = 3). Significant probability is shown as *P ≤ 0.05 (SEM)

At 24 h post-PBM, a significant increase in total GSK3β was noted in DW cells (*P = 0.05)*, with a large effect size (d_Cohen_ of 4.1), and a significant decrease in N and DHW cell models (*P = 0.05)* with large effect sizes (d_Cohen_ of 2.1 and 1.8, respectively) (Fig. 7a). Insignificant changes were observed at 48 h (Fig. 7b).

Comparisons between non-irradiated N cells and non-irradiated and irradiated W, D, DW, and DHW cells 24 h post-PBM were made. An observable decrease in GSK3β was observed in non-irradiated D (*P = 0.015)* and DW *(P = 0.009)* cell models, Similarly, comparisons were made between groups at 48 h post-PBM, no significant changes were observed.

**Fig. 8:**
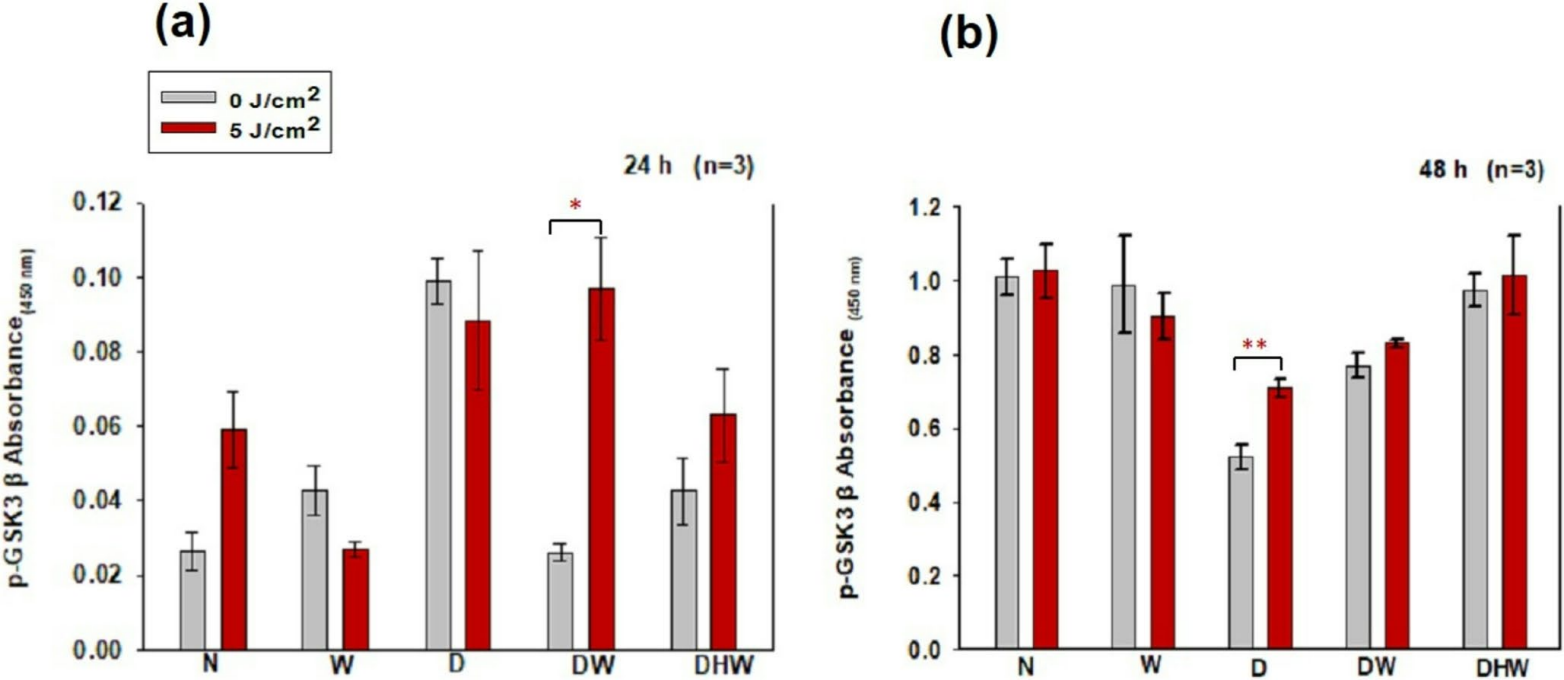
Phosphorylated (p-) GSK3β in normal (N), wounded (W), hyperglycemic (D), hyperglycemic wounded (DW), and hyperglycemic hypoxic wounded (DHW) cell models 24 h (**a**) and 48 (h) (**b**) post-PBM at 830 nm with 5 J/cm^2^ (*n* = 3). Significant probability is shown as *P ≤ 0.05 (SEM) and P < 0.01** (SEM)

There was a significant increase in p-GSK3β in DW cell models (*P < 0.05*) at 24 h post-PBM with a large effect size (d_Cohen_ of 7.0) (Fig. 8a). A significant increase was observed in D cell models *(P < 0.001)* with a large effect size (d_Cohen_ of 3.8) at 48 h (Fig. 8b).

Non-irradiated N cell models were compared to non-irradiated and irradiated W, D, DW, and DHW cell models at 24 h post-PBM. A significant increase in p-GSK3β was observed in non-irradiated D cells *(P = 0.001*), while no observable change was seen in W, D, and DHW cells and an increase in irradiated DW (*P = 0.009).* At 48 h, a significant decrease in p-GSK3β was observed in non-irradiated D (*P = 0.001)* and DW *(P = 0.015)* cell models, with no observable changes in W and DHW cells while a decrease was seen in irradiated D *(P = 0.005)* and DW *(P = 0.023)* with no significant changes in W and DHW.

## Discussion and conclusion

PBM has been used in wound healing to alter physiological processes and induce therapeutic effects, thereby speeding up the process. Wound healing involves various cellular and molecular processes, as well several growth factors like VEGF and signalling pathways such as PI3K/AKT/mTOR. PBM in the red spectrum (660 nm with 5 J/cm^2^) was found to stimulate the release of growth factors and activate several signalling pathways essential for wound healing [18;19], including VEGF and the PI3K/AKT/mTOR signalling pathway [20]. The purpose of this study was to ascertain whether PBM in the NIR spectrum, at 830 nm with a fluence of 5 J/cm^2^, affected the PI3K/AKT/mTOR signalling pathway and downstream cellular processes (cellular migration, proliferation, and survival) in a hyperglycemic, wounded human skin fibroblast cell model. Fibroblast cells are key players in wound healing [30]. This study was conducted on five fibroblast cell models, namely normal (N), wounded (W), hyperglycemic (D), hyperglycemic wounded (DW), and hyperglycemic hypoxic wounded (DHW). The cell models used in the present study and a fluence of 5 J/cm^2^ are well-established and have been used in similar PBM studies conducted by our research group [18, 23, 24, 31–33].

Several analytical techniques were applied to both non-irradiated (control) and irradiated skin fibroblast cells at 24 and 48 h post-PBM to assess cellular morphology/migration, viability, proliferation, and survival, and the activation of proteins linked to the PI3K/AKT/mTOR signalling pathway. According to studies conducted in-*vivo* and in-*vitro*, PBM promoted collagen synthesis, growth factor production, and fibroblast and keratinocyte cell migration to speed up the wound healing process [34, 35]. Numerous cell signalling pathways have been demonstrated to be activated by PBM.

Cellular morphology and cellular migration rate towards the central scratch, or ‘wound’, in both non-irradiated and irradiated cell models was analysed. All cell models maintained their characteristic morphology throughout the experiments at both 24 and 48 h post-PBM. In wounded cell models (W, DW, and DHW), a significant increase in the number of cells around the cell free zone boundaries were noted. The central scratch in irradiated models was completely covered with new cells at a faster rate than control models. Complete wound closure was observed at 48 h in all irradiated wounded cell models, including cells grown under hyperglycemic and hypoxic conditions, while gaps were still present the controls. An observable increase in cellular migration rate occurred 24 h post-PBM in irradiated W and DHW models, and at 48 h in DW and DHW models. Hyperglycemia may hinder wound healing by altering fibroblast cellular migration [36], however, this process is restored and even increased when treated with PBM at 830 nm with 5 J/cm^2^. Results from this study correlate with the results obtained by other studies making use of 660 nm [18, 19, 32] and 830 nm [24] with 5 J/cm^2^.

Delayed wound healing has been associated with several factors including abnormal fibroblast migration, proliferation, differentiation, and apoptosis. Several research papers suggest that hypoxia may be associated with increased apoptosis and decreased Bcl-2. Cellular viability in this study was assessed using the Trypan blue exclusion assay, while cellular apoptosis was determined through Annexin V/PI staining and measurement of the anti-apoptotic protein Bcl-2. PBM at 830 nm with 5 J/cm^2^ resulted in a significant increase in viability at 24 h in N, D, DW, and DHW cell models, and in D, DW, and DHW cell models at 48 h. These results correspond with the apoptosis findings. The annexin V/PI results showed that most of the cell population remains viable post-PBM. Although there was a slight significant increase in necrosis in DHW cells 24 h post-PBM (1.4%), most cells remained viable (80%). At 48 h there was a significant increase in the percentage of viable cells in the DHW models, with 97% viable cells compared to 78% in the control. No significant increases in early or late apoptosis were detected. There was a significant increase in Bcl-2 24 h post-PBM in N, W, D, and DHW cell models, and in N, D, and DHW cell models at 48 h post-PBM. Although a decrease was observed in DW models at both 24 and 48 h post-PBM, most cells remained viable as indicated by the Annexin V/PI results. This study suggests that PBM at 830 nm with 5 J/cm^2^ promotes cellular viability and reduces apoptosis in cells grown under hyperglycaemia and hypoxic conditions. These results agree with the study findings obtained by Jere et al. (2018) [19] and Ayuk *et al. (*2018) [25], whereby PBM at 660 nm with a fluence of 5 J/cm^2^ increased viability of hyperglycemic wounded cells. Faria et al., (2020) [37] found that PBM with 830 nm, (power output of 10 mW, fluence of 3 and 30 J/cm^2^ irradiated in a continuous emission mode, and exposure time of 15 and 150 s) for four consecutive days altered Bcl-2 mRNA levels, caspase-6 and Bcl-2 protein levels, modulating cell survival and reducing DNA fragmentation. A recent study by Mutafchieva and colleagues [38] showed that PBM promoted the expression of anti-apoptotic (Bcl-2) and proliferative (Ki-67) markers, suggesting that this type of therapy promotes the regeneration of the damaged tissue.

Diabetes is linked to numerous pathological changes that contribute to poor wound healing. Excessive inflammation, reduced angiogenesis, keratinocyte migration disruption, and decreased fibroblast proliferation are characteristic of hyperglycemic wounds [39]. Due to increased glucose-stimulated oxidative stress, hyperglycaemia slows down the proliferation and motility of fibroblast cells, which results in delayed wound healing [40]. In this study cellular proliferation was assessed using BrdU incorporation and analysed using flow cytometry. Cytometric investigation of the cell cycle demonstrated that PBM at 830 nm and 5 J/cm^2^ increased cellular proliferation as seen by the decrease in the population of cells in the resting phase, and the increase in the number of cells in the synthesis and mitotic phase. At 48 h post-PBM, the highest population was observed in the resting phase, having moved through the synthesis and mitotic phases prior to this. The literature clarifies that PBM has very little or no effect on normal, unstressed cells [14].

These results correlate with the results obtained in a study conducted by Jere and colleagues (2020) [33], whereby cells were irradiated at a wavelength of 660 nm and a fluence of 5 J/cm^2^. The current study also aligns with a study conducted by Oyebode and Houreld (2022) [24], in which BrdU incorporation was used to assess cellular proliferation. The cells were irradiated using the same parameters as the present study, and PBM showed changes following irradiation at 24 and 48 h. The present study did not show many changes at 24 h as compared to the changes observed at 48 h which indicated the cells’ ongoing activity. Although there is a decrease in irradiated W cells in the S phase at 24 h, there is only a small population of cells in this phase (4%), with a large percentage of cells already in the mitotic phase (G2/M).

Delayed wound healing in patients with DM has been associated with several factors, including abnormal fibroblast migration, proliferation, differentiation, and apoptosis, as well as hypoxia [41]. Numerous biological processes essential for wound healing depend on oxygen. Proper oxygen supply to the injured tissue affects the healing process and the outcomes of different therapeutical approaches [42, 43]. The secretion of growth factors by various cells in the ECM is a crucial component of wound healing and a primary contributor to the process. PBM promotes wound healing by stimulating the body’s production of growth factors that support tissue repair and regeneration [5, 18, 44–46].

Numerous cell types contribute to wound healing, including fibroblasts, which produce VEGF. VEGF plays a crucial role in wound healing. When the VEGF-receptor pathway is activated, it initiates a series of signaling events that promote cell growth, migration, and survival of endothelial cells derived from existing blood vessels [47]. DM lowers cellular signalling, including the PI3K/AKT signalling pathway that plays a crucial role in wound healing. PI3K converts phosphatidylinositol 4,5-bisphosphate (PIP2) to phosphatidylinositol 3,4,5- triphosphate (PIP3), which then attracts AKT to the membrane where PIP3 is phosphorylated by phosphoinositide-dependent kinase 1 (PDK1). Activated AKT is responsible for the initiation of various downstream proteins, including mTOR and GSK3. mTOR is involved in cellular proliferation, migration, angiogenesis, and survival and significantly boosts cutaneous wound healing [48, 49]. Hypoxia stimulates cellular mTOR activity, whereas persistent hypoxia causes its inhibition [50].

GSK3β is essential for several physiological functions, including gene expression, glycogen metabolism, and cellular signalling. It is also a major regulator of several conditions, including diabetes [51]. The activity of GSK-3*β* can be reduced by phosphorylation at Ser-9. GSK3*β* is an active form of the protein, while the phosphorylated form of GSK3*β* is inactive. GSK-3β also plays a role in cell cycle regulation by phosphorylating cyclin D1, which causes the protein to be rapidly broken down by proteases [52]. Inhibition of GSK3β activity leads to stabilisation of β-catenin, a main effector in the Wnt signalling pathway. This stabilisation allows β-catenin to accumulate in the cytoplasm which is translocated into the nucleus where it functions to regulate gene expression and promotes cell proliferation, differentiation and survival. Therefore, an increase in p-GSK3B means a decrease in apoptosis and increase in cell survival.

In this study, VEGF levels and the activation of proteins involved in the PI3K/AKT pathway (PI3K, p-AKT, p-mTOR, GSK3β, and p- GSK3β) were determined 24 and 48 h post-PBM with 830 nm and 5 J/cm^2^. No significant changes were observed in VEGF post-PBM, except for an observable decrease seen in the DHW cell model at 24 h. However, this decrease was no longer evident at 48 h post-PBM. VEGF levels increased in all models (control and experimental) and were higher at 48 h. PBM at a wavelength of 660 nm has been consistently reported to enhance wound healing through the stimulation of VEGF, which promotes angiogenesis and tissue repair [53]. This effect is generally due to the absorption of red light by cytochrome c oxidase, resulting in increased mitochondrial activity and transcriptional activation of VEGF [54]. In contrast, the current study did not show any significant change in VEGF. This discrepancy may be due to different wavelengths and fluencies, different cell models and cell types, and even different times the protein is measured post-PBM. This shows that PBM is highly adaptive rather than uniform. Instead of being inconsistent, PBM is a flexible tool whose results vary depending on experimental parameters, allowing it to be tailored to specific therapeutic goals. While light at 660 nm appears to increase VEGF transcription, near-infrared light at 830 nm may change the dynamics of reactive oxygen species, nitric oxide production, or transcription factor activity in ways that restrict VEGF gene expression. Alternatively, a wavelength of 830 nm might promote healing via VEGF-independent processes, such as collagen deposition, fibroblast differentiation, or inflammatory pathway modulation [55–59]. Western blotting revealed no positive bands for PI3K, nor for activated p-AKT and p-mTOR post-PBM. GSK3β (active form) was significantly increased in the DW cell model at 24 h post-PBM; however, this did not result in a significant decrease in cell viability, as evidenced by results from the Trypan blue exclusion assay and Annexin V/PI staining, which showed that 71% of DW cells remained viable. A significant decrease in GSK3β was observed in the N and DHW cell models at 24 h post-PBM. The inactive form of GSK3β (p-GSK3β) significantly increased in the DW cell model at 24 h, and in the D cell model at 48 h. Both GSK3β and p-GSK3β levels increased with time, with higher levels in all models at 48 h compared to 24 h.

According to the findings of this study, the PI3K/AKT signalling pathway was not activated by VEGF, and PBM at a wavelength of 830 nm and a fluence of 5 J/cm^2^ did not affect the PI3K/AKT/mTOR signalling pathway. The effects of PBM on cellular proliferation, viability, motility, and survival is likely due to activation of other signalling pathways. These results contradict a similar research conducted by Jere et al.., (2022) [20] on the same cell models using a wavelength of 660 nm with the same fluence of 5 J/cm^2^. Their study reported increased VEGF levels and activation of the PI3K/AKT/mTOR pathway. Another study conducted by Tian and colleagues (2023) [58] revealed that PBM at 830 nm and a fluence of 5 J/cm^2^ reduced mRNA expression of iNOS, IL-12, TNF-α, IL-1β, and IL-6, and increased the expression of Arg1 and IL-10 by M1-type macrophages via the PI3K/AKT/mTOR pathway. Another study conducted in African green monkey (SV40) by Zhang and colleagues (2009) [60] demonstrated that PBM at a wavelength of 632.8 nm and a fluence of 1.2 J/cm^2^ promoted cellular proliferation via the PI3K/Akt signalling pathway. The present study found that PBM at 830 nm with 5 J/cm^2^ (power density 11 mW/cm^2^) did not activate the PI3K/AKT/mTOR signalling pathway or VEGF release in a hyperglycemic wounded cell model; suggesting that other signalling pathways may be involved, such as Wnt/β-catenin. This signalling pathway regulates transcriptional programs that control proliferation, differentiation, and stemness. Additionally, it can also drive the G1-S transition via Cyclin D/Cyclin-Dependent Kinase (CDK) activation and repression mechanisms [61]. Similar to this, when PI3K signalling is suppressed, the mitogen-activated protein kinase (MAPK) cascade like extracellular signal-regulated kinase (ERK), c-Jun N-terminal kinase (JNK), and p38 mitogen-activated protein kinase (p38 MAPK) can sustain growth or stress adaptation independently of AKT by translating extracellular and stress signals into proliferation, differentiation, apoptosis or senescence [51]. Concurrently, Nuclear Factor Erythroid 2–Related Factor 2 (Nrf2) coordinates an antioxidant response by upregulating detoxifying enzymes like HO-1 and NQO1, thereby reducing ROS, safeguarding mitochondria, and promoting metabolic flexibility, which provides survival outcomes and resilience against oxidative stress without directly involving mTOR [62]. Beyond these, further compensatory mechanisms are provided by stress response mediators such as AMP-Activated Protein Kinase (AMPK), the Unfolded Protein Response (UPR) pathways, Protein Kinase R-like Endoplasmic Reticulum Kinase (PERK)/Activating Transcription Factor 4 (ATF4), Inositol-Requiring Enzyme 1 (IRE1)/X-box Binding Protein 1 (XBP1), Activating Transcription Factor 6 (ATF6), autophagy, Nuclear Factor kappa-light-chain-enhancer of activated B cells (NF-κB), and Tumor Protein p53 (p53)/c-Jun N-terminal Kinase (JNK), which allow cells to respond to DNA damage, proteotoxic stress, or nutritional deprivation without the assistance of AKT/mTOR. Together, these pathways reveal a complex network of signalling interactions, in which Wnt may promote proliferation, Nrf2 reduces oxidative burden, ERK promotes growth, AMPK and UPR buffer metabolic stress, and NF-κB modulates inflammation and survival, underscoring the need to profile parallel axes when PI3K/AKT/mTOR appears unnecessary. Therefore, this integrated view suggests that the phenotype observed is likely the result of coordinated activity across multiple pathways, rather than dependence on a single signalling pathway.

The present study shows that PBM at 830 nm promotes the process of wound healing in fibroblasts models through enhanced viability, migration and proliferation, without activating the PI3K/AKT/mTOR pathway or increasing the secretion of VEGF at the timeframes studied and laser parameters used (5 J/cm^2^). This contrasts previous results obtained at a wavelength of 660 nm with 5 J/cm^2^ [20]. The differential VEGF responses suggest that signalling may vary in a wavelength-specific manner, with 830 nm engaging alternative transcriptional programs, such as Nrf2-mediated stress adaptation or NF-κB modulation [63, 64], rather than the 660 nm light-induced HIF-1α/VEGF axis [65, 66]. A key component of PBM photobiology is the concept of wavelength-dependent pathway activation [67–69]. Therefore, this suggest that the therapeutic mechanism of PBM are dependent on the selected parameters with 830 nm and 5 J/cm^2^ promoting wound repair through pathways that emphasize cellular resilience and remodeling of the matrix instead of angiogenesis. Since multiple signalling pathways are involved in wound healing, further studies at different wavelengths and fluencies are required to investigate these pathways in order to gain a deeper understanding of the underlying mechanisms of PBM.

## Study limitation and future recommendations

The current study provides valuable insight into the function of a single cell type in wound healing, however, a few study limitations should be acknowledged. Wound healing is a complex, multicellular process that requires the coordinated action of diverse cell populations. The absence of multicellular interactions in this study means that key processes such as angiogenesis, inflammation, and matrix remodeling were not fully captured, which may have led to either an overestimation or underestimation of the observed effects. Additionally, only one fluence was investigated, limiting the ability to assess dose dependent responses, leaving open the possibility of the biphasic effect. The PI3K/AKT/mTOR pathway is a fast-response signalling cascade, activated within minutes to hours. Capturing its early dynamics requires a comprehensive time-series analysis. To fully characterize the pathway’s activation profile, future research should incorporate earlier time-point measurements, e.g. 6–12 h. Therefore, to enhance translational relevance, subsequent research should employ more complex systems that better replicate the structural and functional complexity of wound healing, such as co-culture models, organoids, or 3D scaffolds.

## Supplementary Information

Below is the link to the electronic supplementary material.

Supplementary Material 1 (JPG 83.7 KB)

Supplementary Material 2 (JPG 31.1 KB)

Supplementary Material 3 (JPG 470 KB)

Supplementary Material 4 (JPG 46.4 KB)

Supplementary Material 5 (JPG 36.4 KB)

Supplementary Material 6 (JPG 564 KB)

Supplementary Material 7 (JPG 78.5 KB)

Supplementary Material 8 (JPG 387 KB)

Supplementary Material 9 (JPEG 15.6 KB)

Supplementary Material 10 (JPG 556 KB)

Supplementary Material 11 (JPG 95.1 KB)

Supplementary Material 12 (JPG 542 KB)

Supplementary Material 13 (JPG 709 KB)

## Data Availability

The authors declare that the data supporting the findings of this study are available within the paper. Should any raw data files be needed in another format they are available from the corresponding author upon reasonable request.
