# Supplementary figures and images for: Photobiomodulation at 830 nm with 5 J/cm2 does not promote PI3K/AKT/mTOR signalling pathway activation in hyperglycemic wounded cells
Source: Lasers Med Sci. 2026 Mar 27;41(1):60. doi: 10.1007/s10103-026-04859-8 (PMC13021734; doi:10.1007/s10103-026-04859-8)

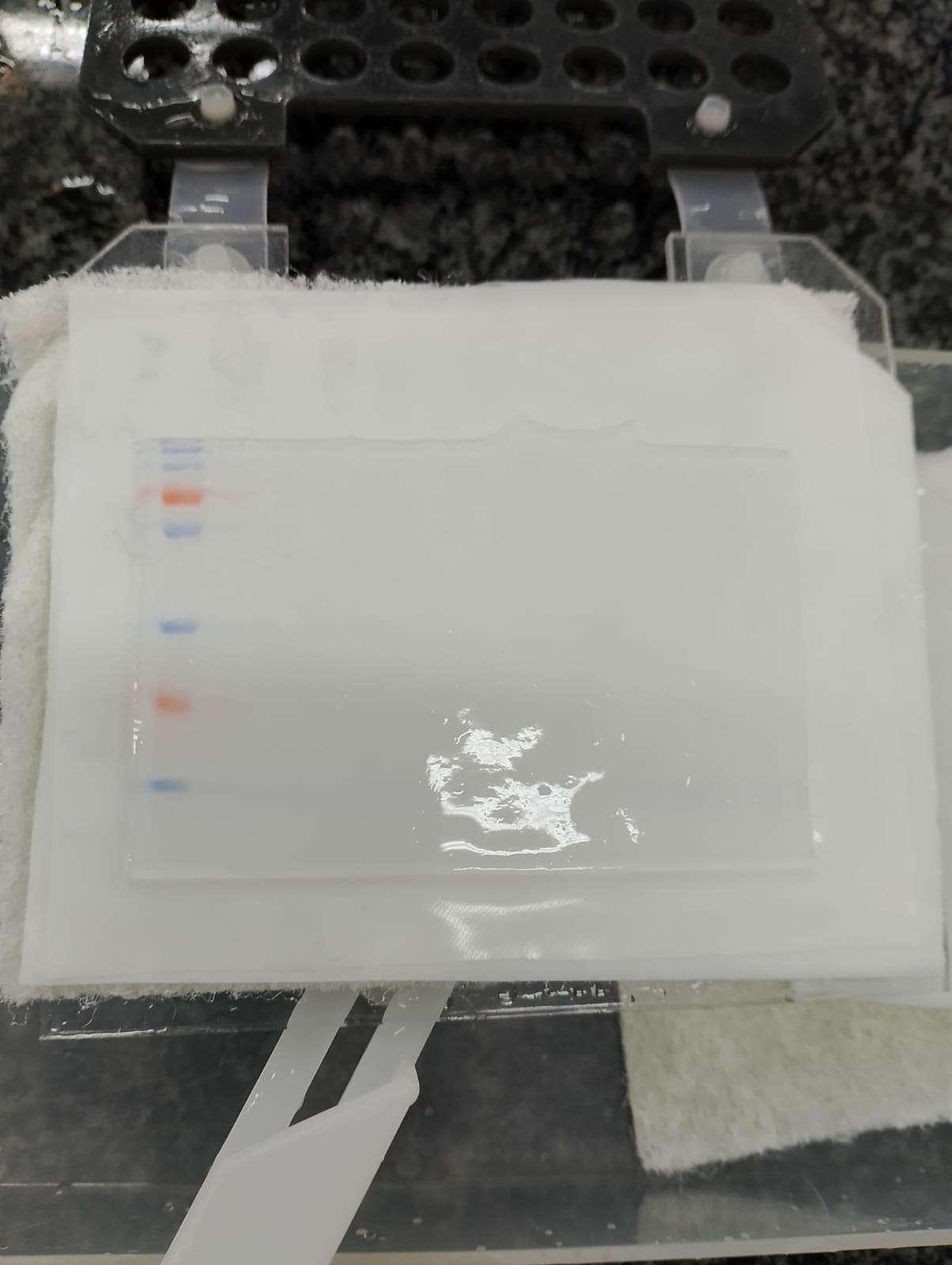

Supplement: Supplementary file 1 — Supplementary Material 1 (JPG 83.7 KB) [file 10103_2026_4859_MOESM1_ESM.jpg]

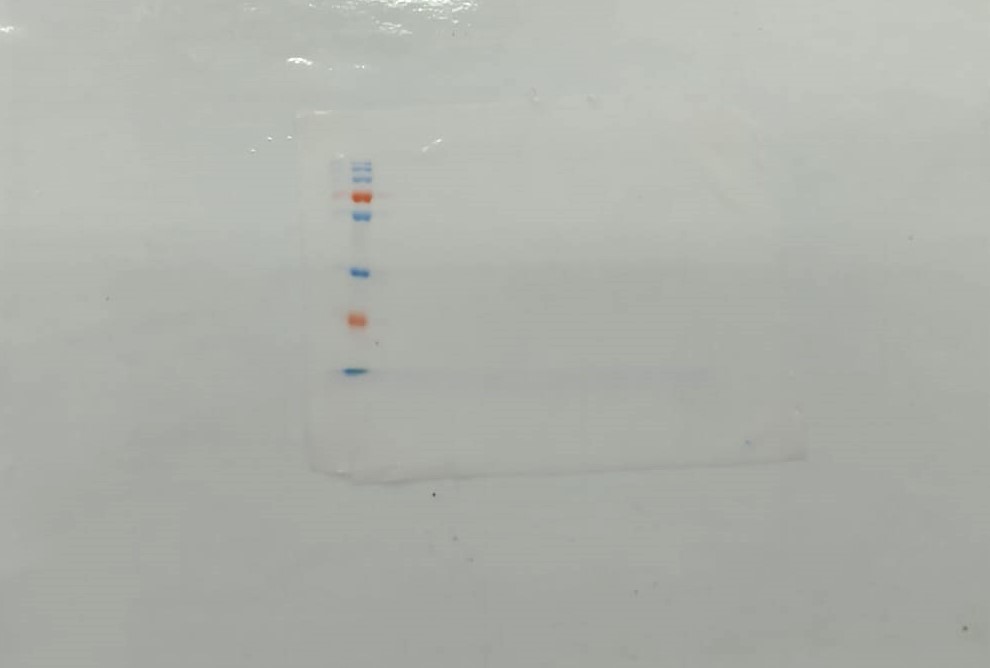

Supplement: Supplementary file 2 — Supplementary Material 2 (JPG 31.1 KB) [file 10103_2026_4859_MOESM2_ESM.jpg]

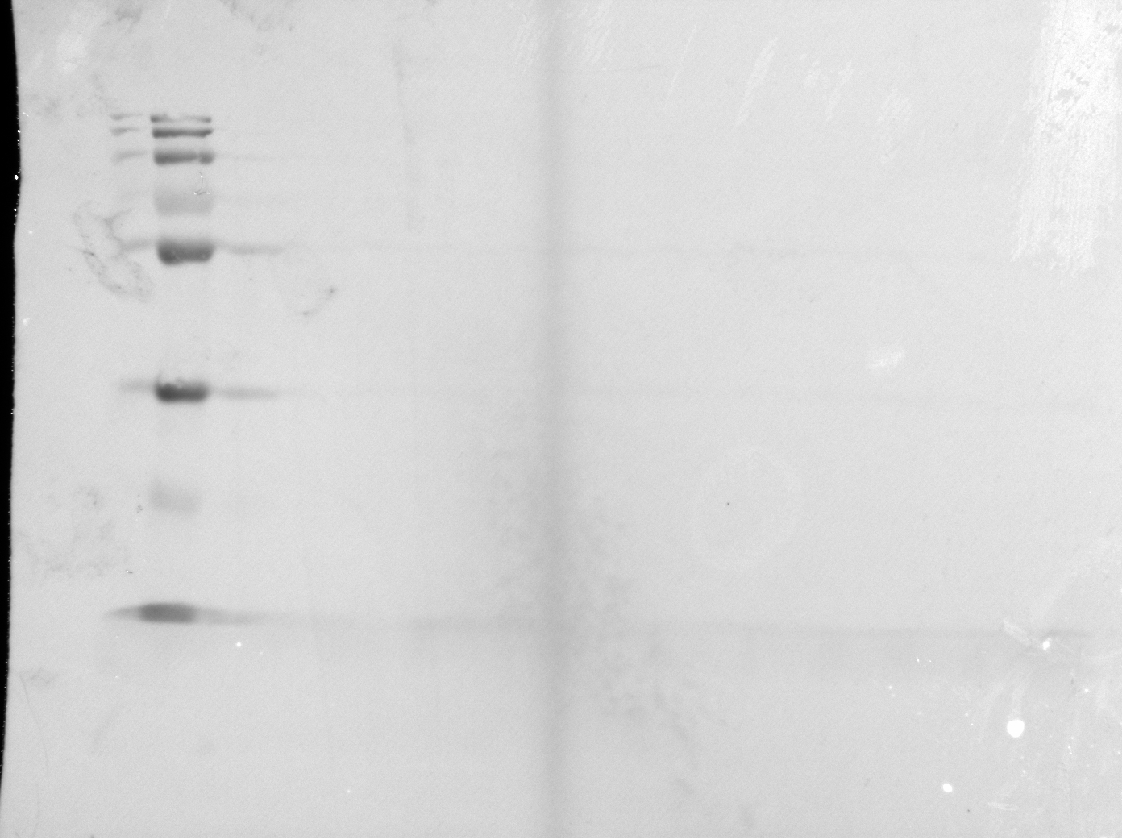

Supplement: Supplementary file 3 — Supplementary Material 3 (JPG 470 KB) [file 10103_2026_4859_MOESM3_ESM.jpg]

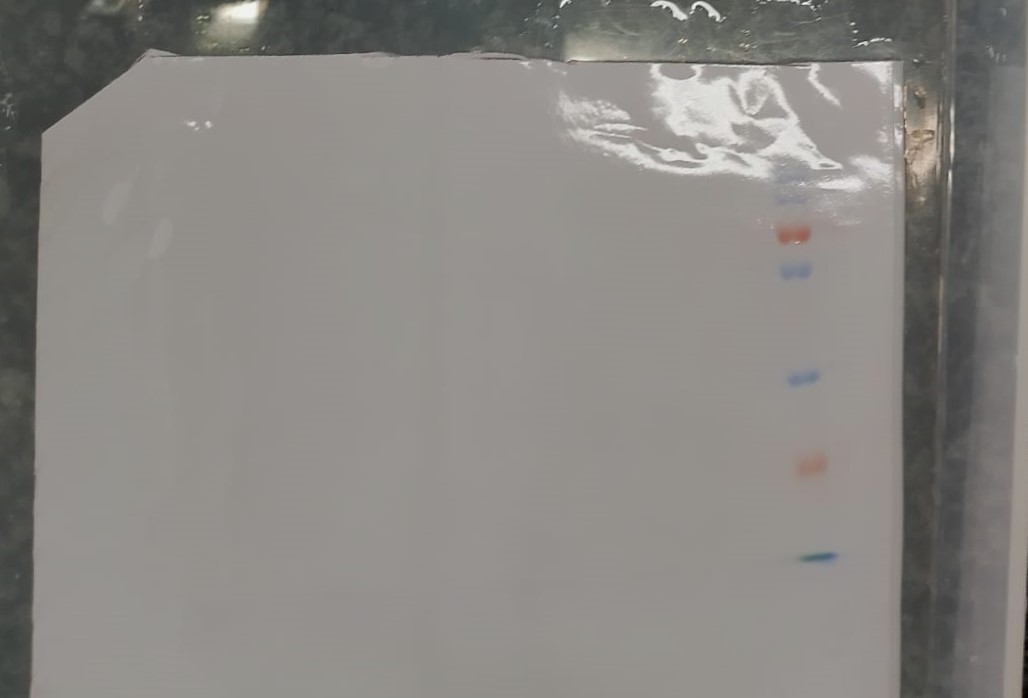

Supplement: Supplementary file 4 — Supplementary Material 4 (JPG 46.4 KB) [file 10103_2026_4859_MOESM4_ESM.jpg]

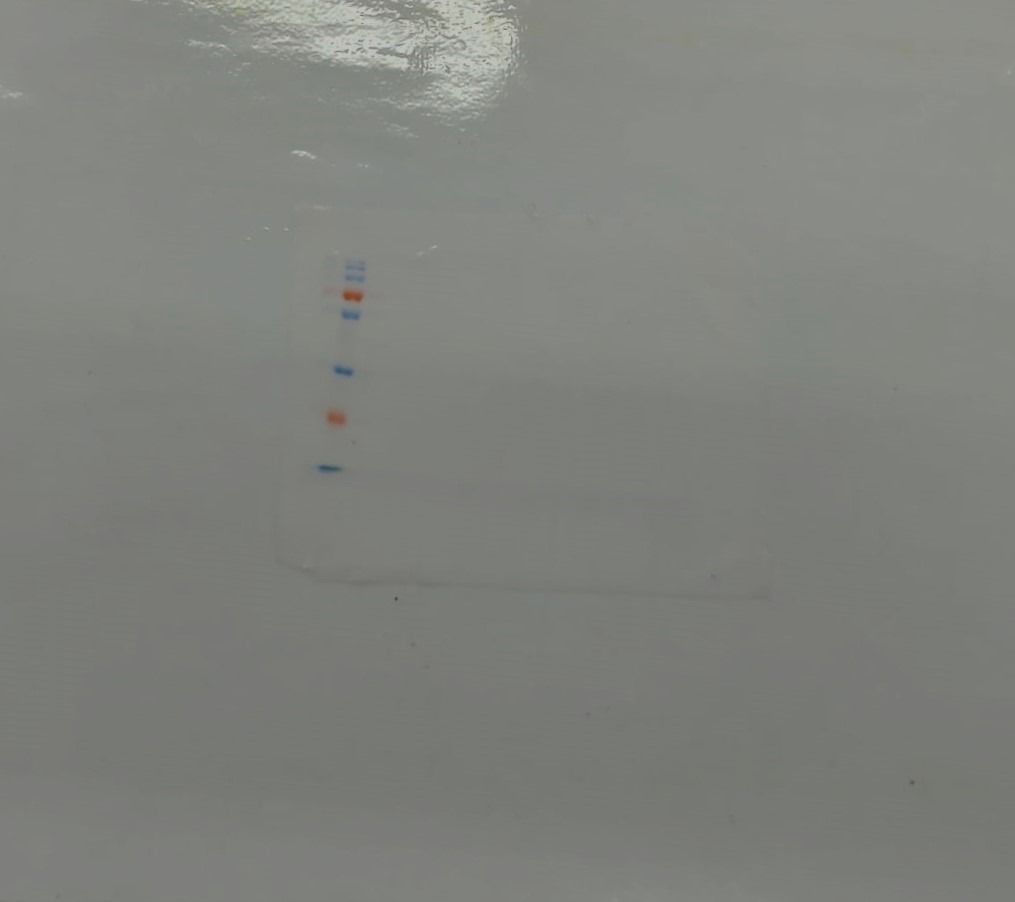

Supplement: Supplementary file 5 — Supplementary Material 5 (JPG 36.4 KB) [file 10103_2026_4859_MOESM5_ESM.jpg]

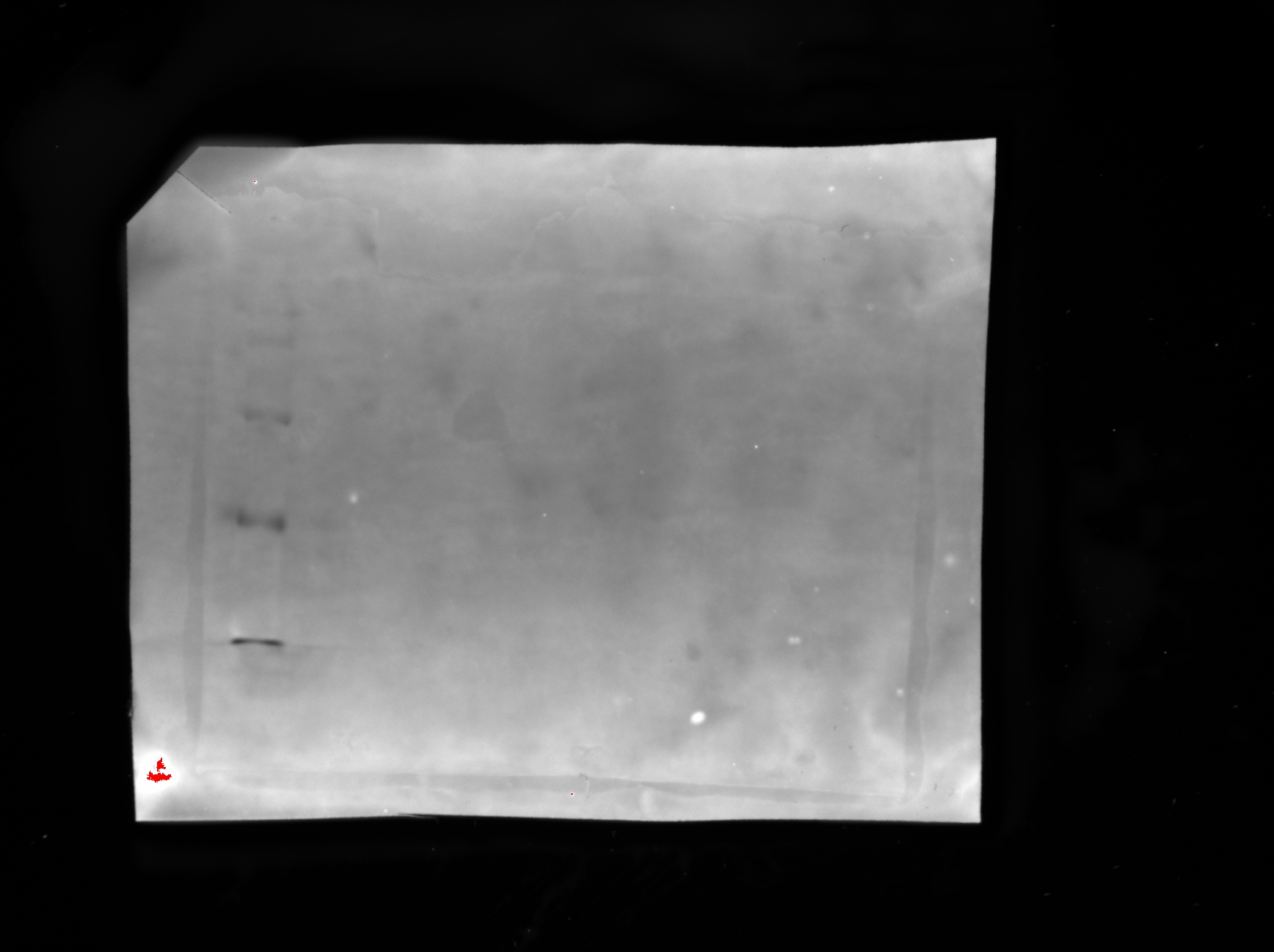

Supplement: Supplementary file 6 — Supplementary Material 6 (JPG 564 KB) [file 10103_2026_4859_MOESM6_ESM.jpg]

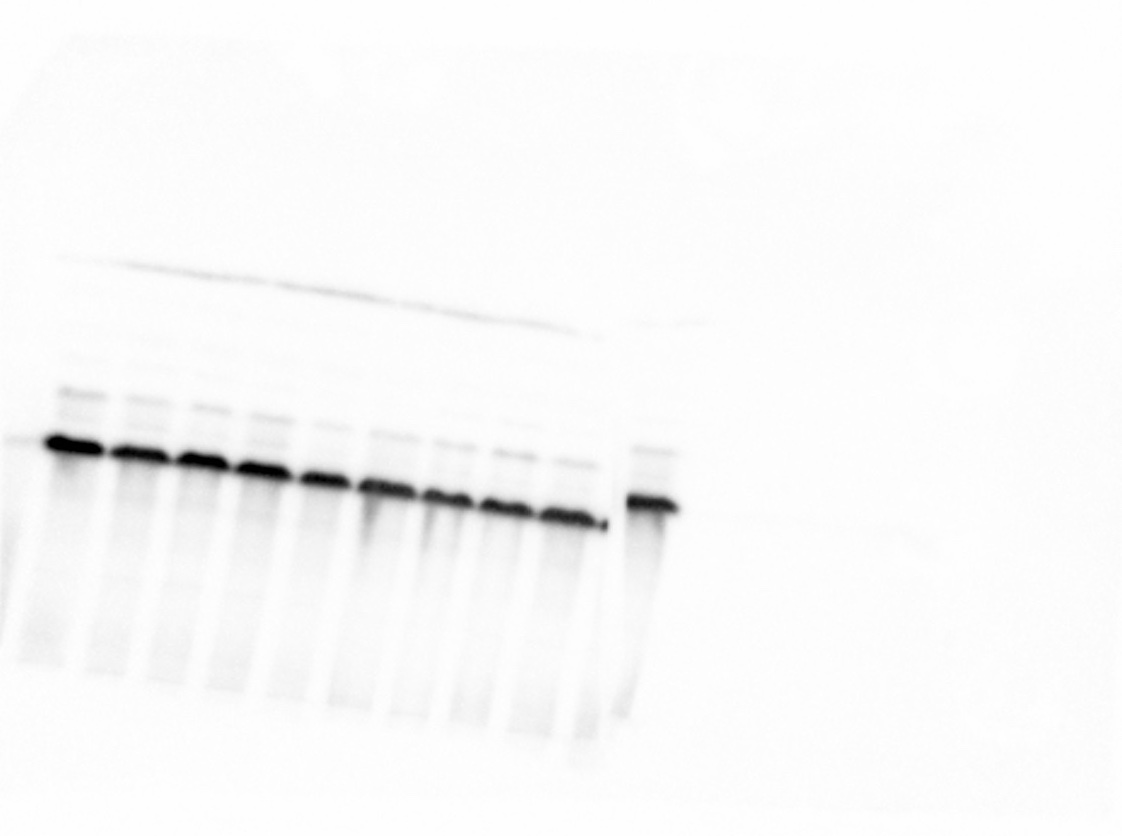

Supplement: Supplementary file 7 — Supplementary Material 7 (JPG 78.5 KB) [file 10103_2026_4859_MOESM7_ESM.jpg]

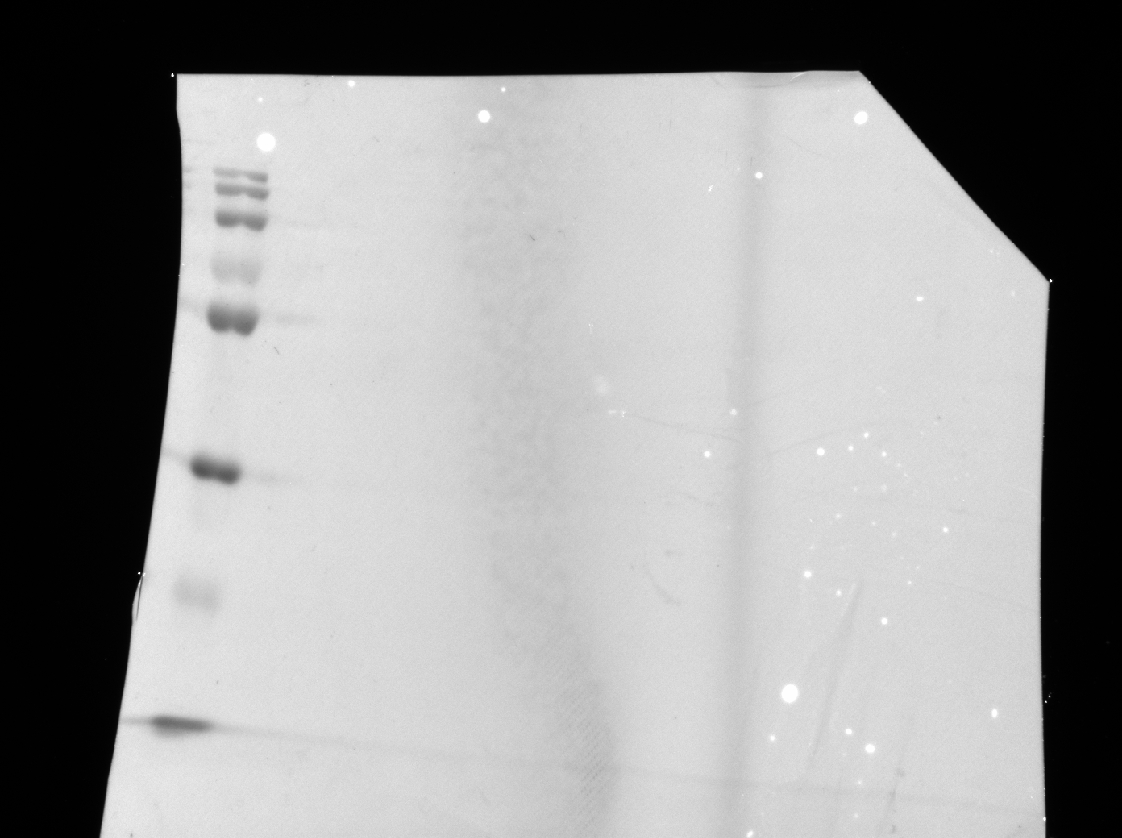

Supplement: Supplementary file 8 — Supplementary Material 8 (JPG 387 KB) [file 10103_2026_4859_MOESM8_ESM.jpg]

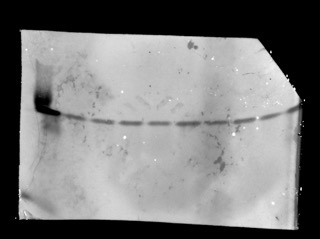

Supplement: Supplementary file 9 — Supplementary Material 9 (JPEG 15.6 KB) [file 10103_2026_4859_MOESM9_ESM.jpeg]

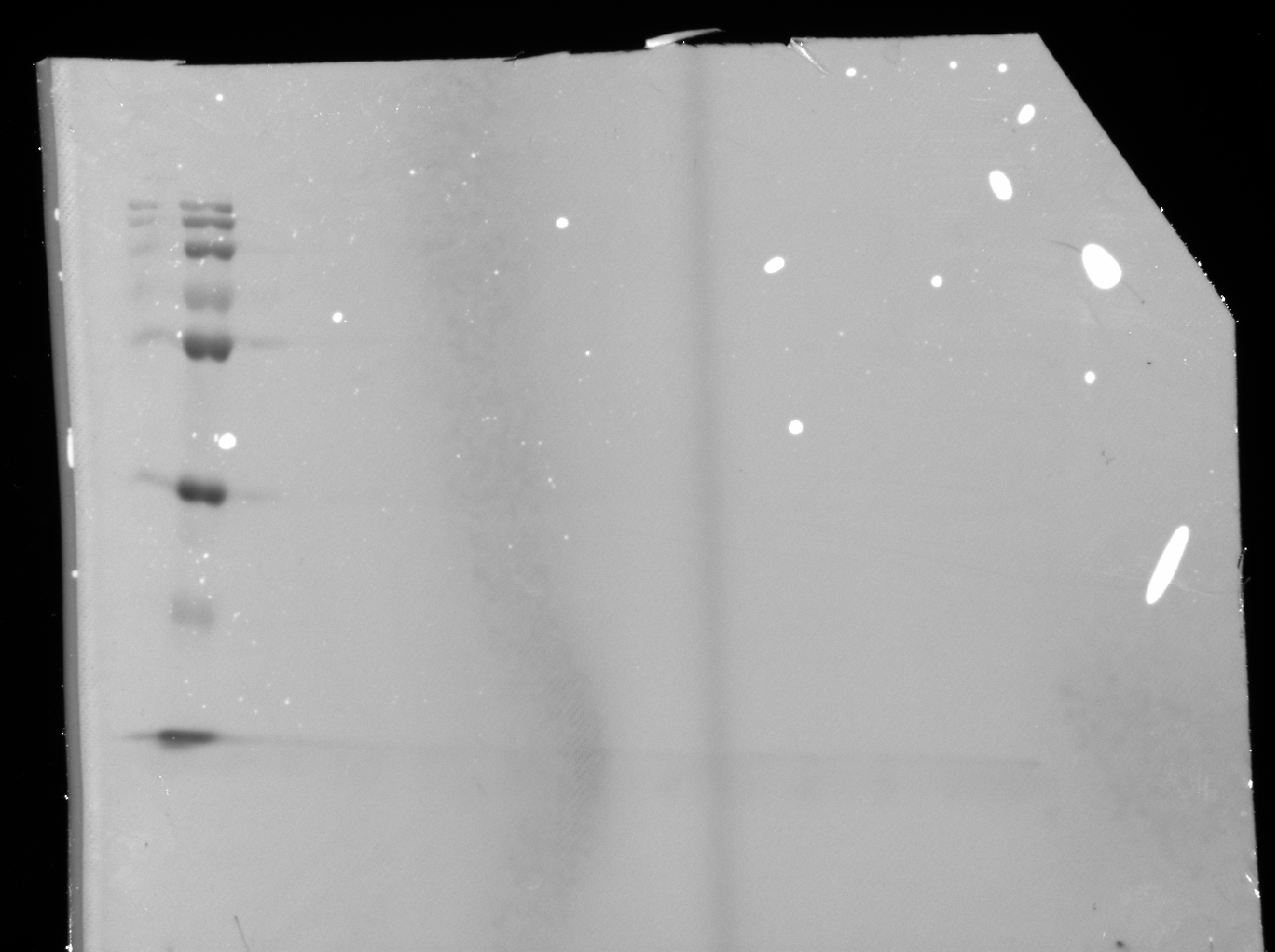

Supplement: Supplementary file 10 — Supplementary Material 10 (JPG 556 KB) [file 10103_2026_4859_MOESM10_ESM.jpg]

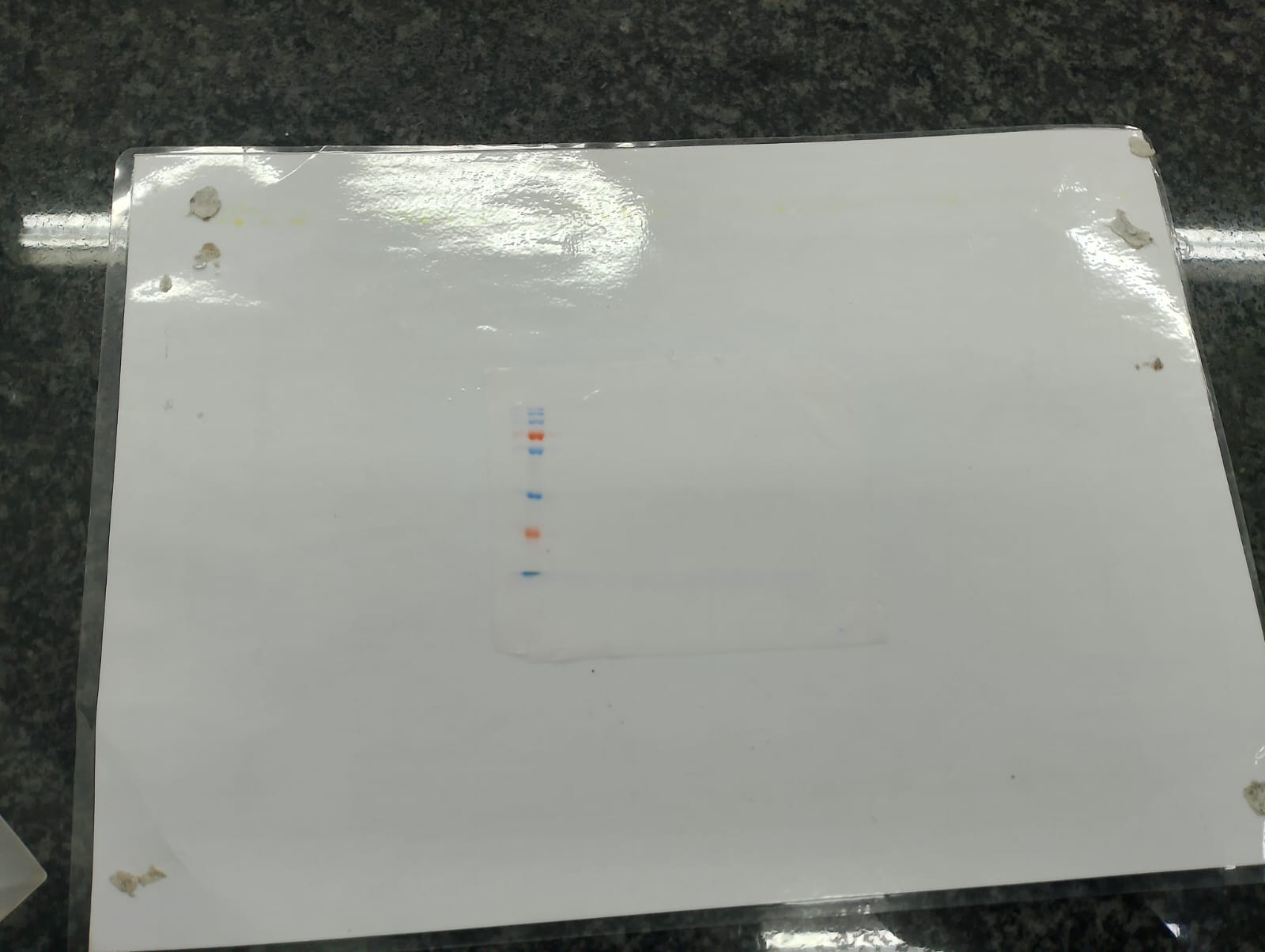

Supplement: Supplementary file 11 — Supplementary Material 11 (JPG 95.1 KB) [file 10103_2026_4859_MOESM11_ESM.jpg]

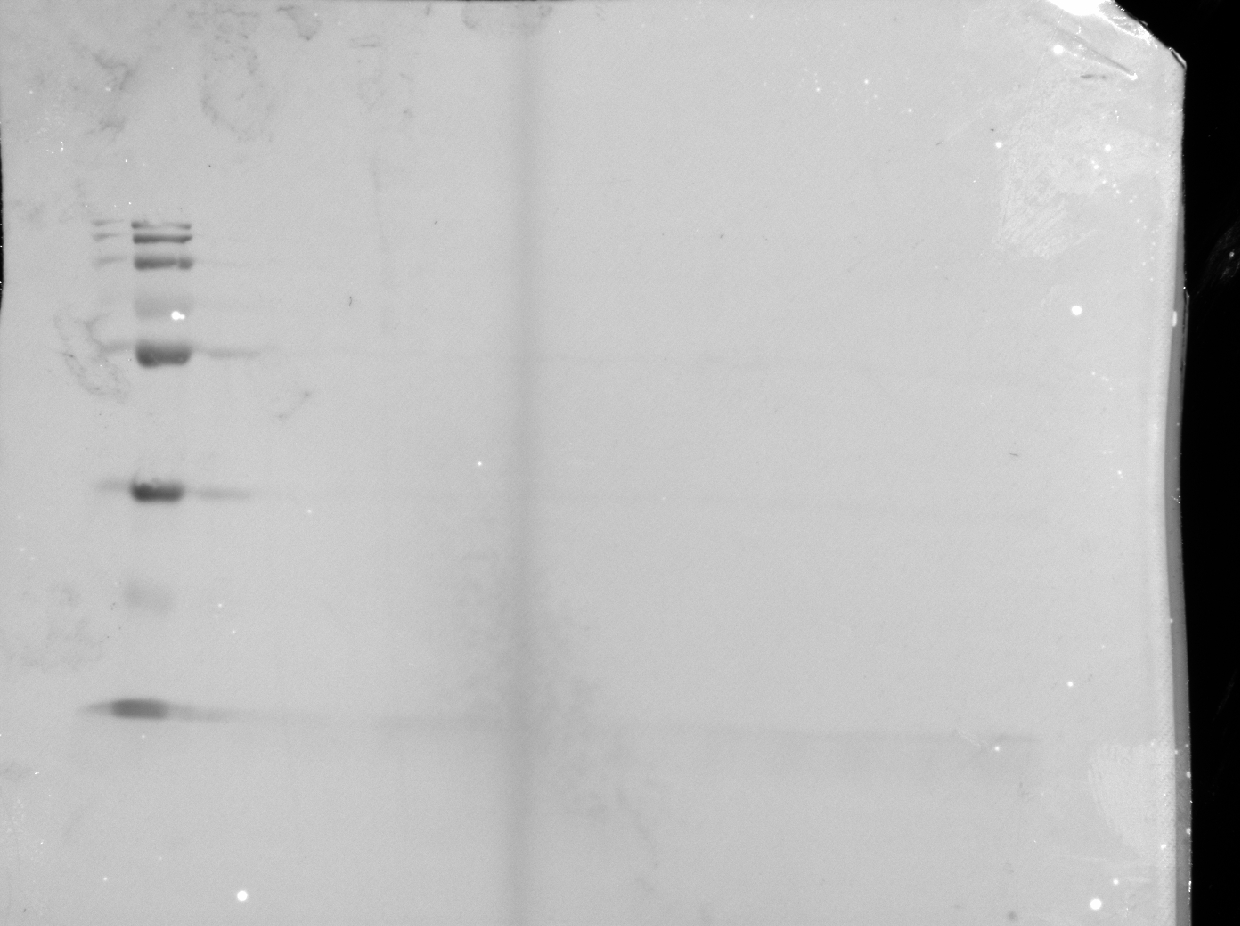

Supplement: Supplementary file 12 — Supplementary Material 12 (JPG 542 KB) [file 10103_2026_4859_MOESM12_ESM.jpg]

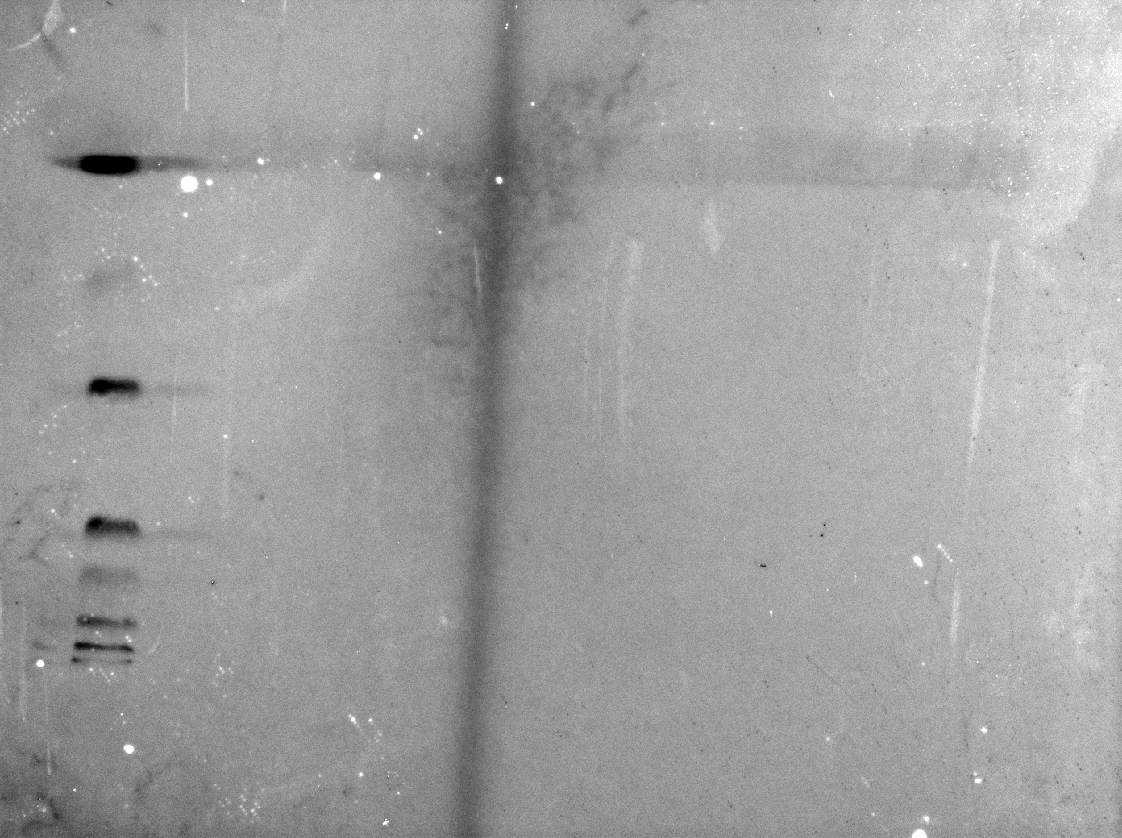

Supplement: Supplementary file 13 — Supplementary Material 13 (JPG 709 KB) [file 10103_2026_4859_MOESM13_ESM.jpg]
